# A Genome-Wide RNAi Screen for Factors Involved in Neuronal Specification in *Caenorhabditis elegans*

**DOI:** 10.1371/journal.pgen.1002109

**Published:** 2011-06-16

**Authors:** Richard J. Poole, Enkelejda Bashllari, Luisa Cochella, Eileen B. Flowers, Oliver Hobert

**Affiliations:** Department of Biochemistry and Molecular Biophysics, Howard Hughes Medical Institute, Columbia University Medical Center, New York, New York, United States of America; Harvard University, United States of America

## Abstract

One of the central goals of developmental neurobiology is to describe and understand the multi-tiered molecular events that control the progression of a fertilized egg to a terminally differentiated neuron. In the nematode *Caenorhabditis elegans*, the progression from egg to terminally differentiated neuron has been visually traced by lineage analysis. For example, the two gustatory neurons ASEL and ASER, a bilaterally symmetric neuron pair that is functionally lateralized, are generated from a fertilized egg through an invariant sequence of 11 cellular cleavages that occur stereotypically along specific cleavage planes. Molecular events that occur along this developmental pathway are only superficially understood. We take here an unbiased, genome-wide approach to identify genes that may act at any stage to ensure the correct differentiation of ASEL. Screening a genome-wide RNAi library that knocks-down 18,179 genes (94% of the genome), we identified 245 genes that affect the development of the ASEL neuron, such that the neuron is either not generated, its fate is converted to that of another cell, or cells from other lineage branches now adopt ASEL fate. We analyze in detail two factors that we identify from this screen: (1) the proneural gene *hlh-14*, which we find to be bilaterally expressed in the ASEL/R lineages despite their asymmetric lineage origins and which we find is required to generate neurons from several lineage branches including the ASE neurons, and (2) the COMPASS histone methyltransferase complex, which we find to be a critical embryonic inducer of ASEL/R asymmetry, acting upstream of the previously identified miRNA *lsy-6*. Our study represents the first comprehensive, genome-wide analysis of a single neuronal cell fate decision. The results of this analysis provide a starting point for future studies that will eventually lead to a more complete understanding of how individual neuronal cell types are generated from a single-cell embryo.

## Author Summary

The generation of a neuron from a fertilized egg requires a multi-step cascade of molecules acting from within and outside that cell to direct it towards a neuronal fate, rather than, say, a muscle cell. These cascades are not fully understood. In this study we systematically eliminate the function of almost all genes in the *C. elegans* genome, one by one, to determine what it takes to build a neuron. We identified 245 genes that affect the development of a specific sensory neuron pair, e.g. the neurons were not generated or the neurons were generated but the terminal fate was not correctly specified. We characterize in more detail the transcription factor *hlh-14*, which we find is required to generate multiple neurons, and the COMPASS histone methyltransferase complex, which we find to have a surprisingly specific role in the specification of a molecular and functional left-right asymmetry in this sensory neuron pair. Our study represents the first genome-wide analysis of a single neuronal cell fate decision. Further characterization of the genes identified here will enhance our understanding, and thus our capacity for treatment and prevention, of human neurological disorders.

## Introduction

When establishing the nematode *C.elegans* as a genetic model system, Sydney Brenner's stated goal was the identification of genes required to build a nervous system [1], [2]. Several decades of investigation, mainly using the classic forward screening methods established by Brenner [1], have indeed revealed genes that are required to generate a diversity of individual neuron types [3]. Yet our understanding of nervous system development still remains spotty. The advent of RNAi technology in *C.elegans* has opened an alternative path to classic forward analysis to study the genetic bases of various processes [4], [5]. However, this approach has not been undertaken in *C.elegans* to comprehensively study neuronal development and fate determination. In this paper we utilize the RNAi approach to screen, at a genome-wide level, for factors involved in all aspects of neuronal development.

**Figure 1:**
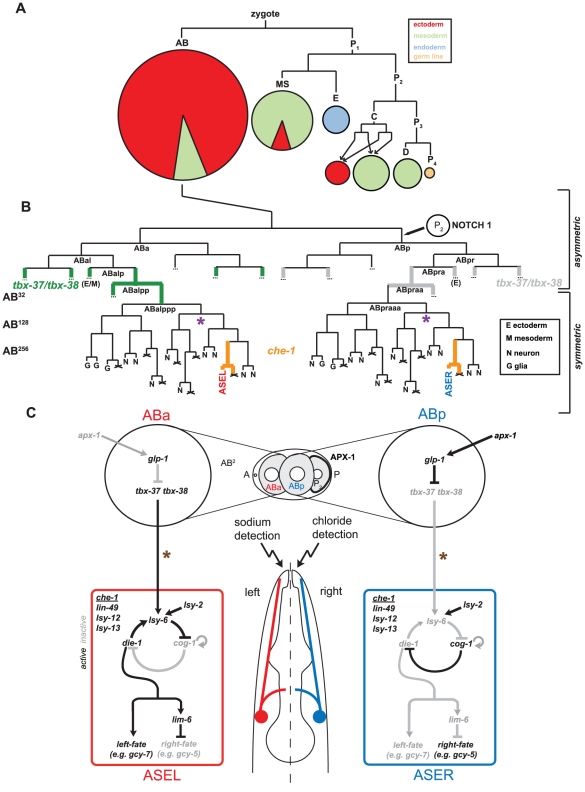
The embryonic origins and specification of the ASE neurons. A: The early lineage of the *C. elegans* embryo leading to the formation of the five founder blastomeres (AB, MS, E, C and P_3_). The pie-charts indicate the relative number of cells and tissue types that are produced from each founder blastomere. The majority of neurons, including ASE, descend form the AB blastomere. B: Lineage of the ASEL and ASER neurons indicating they descend from asymmetric lineage origins which first diverge at the AB^2^-cell stage (adapted from [6]). The Notch signal that specifies the difference between ABa and ABp blastomeres is indicated and leads to the down-regulation of *tbx-37/tbx-38* (expression indicated in green) in the ABp lineage. This early lineage difference is crucial for the specification of ASE left-right asymmetry [12]. The molecular link between the expression of *tbx-37/tbx-38* and the specification of ASE asymmetry is unclear. From the ABalpp/ABpraa stage onwards the lineage produces exclusively ectodermal tissue. From the ABalpppp/ABpraaap stage onwards the lineage produces exclusively neurons, including ASE. Purple asterisks indicate the point at which the proneural gene *hlh-14*, described in this paper, acts to regulate neuronal differentiation in the ASE lineage. C: Summary of the previously described bi-stable feedback loop that regulates ASE left-right asymmetry. The bi-stable feedback loop consists of both transcription factors and micro-RNAs and controls the expression of downstream terminal differentiation genes. All factors in the loop and the downstream terminal factors are regulated in addition by the ASE terminal selector *che-1* (expression indicated in B in orange). The molecular link between the early lineage difference and the bi-stable feedback loop is not known. Brown asterisks indicate that the COMPASS histone-modifying complex, also identified in this paper, acts earlier than *che-1* and before the birth of the ASE neurons to regulate *lsy-6* expression and control ASE asymmetry.

We are using the ASE gustatory neuron class, composed of two bilaterally symmetric neurons, ASEL and ASER, as a paradigm to understand how neuronal fate is specified. Each neuron is produced by two distinct descendants of the embryonic blastomere AB (Figure 1; [6]) called ABa (generates ASEL) and ABp (generates ASER). There is a progressive restriction in the types of cells produced by the AB blastomere. The great-granddaughter of AB, ABalp, still produces cells from two different germ layers (meso- and ectoderm), while one of its daughters, ABalpp only produces neurons and glia. ABalpp's posterior daughter, ABalppp, then becomes committed to only produce neurons, one of them the left ASE neuron. Similar progressive restrictions of fate occur along the ABp lineage that generates the right ASE neuron. Little is known about genes that control these lineage restrictions. The RNAi screen we present here has uncovered a basic helix-loop-helix (bHLH) transcription factor, *hlh-14*, that we characterize in detail in this study. We find *hlh-14* to be crucial for the specification of neurons that derive from several lineage branches, including the ASE neurons.

Once a lineage is committed to only produce neurons (ABalpppp for ASEL and ABpraaap for ASER), fates have to be differentially segregated to generate distinct types of neurons. At this stage, as well as at earlier stages, a binary Wnt-signaling system operates to differentially segregate fates via the anterior-posterior differential distribution of the transcriptional regulators *pop-1* and *sys-1* [7]. These factors presumably interact with stage- and lineage-specific transcription factors to produce distinct cell types [7], [8].

One intriguing aspect of neuronal lineage specification in *C. elegans* is that even though many neurons in the nervous system come as bilaterally symmetric pairs, the lineage of the individual cells that make up a pair can be remarkably distinct [9]. In other words, cells that have limited lineage relation can produce remarkably similar neurons. The pair of ASE neurons falls into this category. As stated above, the left ASE neuron derives from ABa and the right neuron from ABp, which are asymmetrically localized in the early embryo. However, 4 cell divisions later, at the beginning of gastrulation, the ABa and ABp descendants that generate ASEL and ASER (called ABalppp and ABpraaa), begin to undergo left/right symmetric cleavage patterns to generate ASEL and ASER (Figure 1B). During gastrulation the descendants of these cells migrate into left/right bilaterally symmetric positions [10]. How this bilateralization process is regulated at the levels of cell fate and cell morphogenesis is not known.

The correct specification of the ASE neurons involves not only the imposition of ASE neuron identity that is shared by both the left and the right ASE neuron, but also involves the retention of the distinct lineage history of the two neurons. Even though morphologically largely bilaterally symmetric, both ASE neurons express a distinct set of chemosensory receptors and respond differently to distinct sensory cues [11]. The differential, left/right asymmetric expression of these receptors depends on a Notch-mediated signal, received in the early embryo by the lineage that produces ASEL (Figure 1B–1C; [12]). This Notch-mediated signal results in a differential and transient expression of a pair of paralogous T-box genes, *tbx-37/38* [13], and then, several rounds of cell division later, in the expression of the miRNA *lsy-6* in the ASEL, but not the ASER neuron [12]. How the information is transmitted from Notch, via *tbx-37/38*, to *lsy-6* restriction is not known.

The *lsy-6* miRNA has been uncovered through genetic screens for mutants in which the expression of ASEL- and ASER-specific chemoreceptors is disrupted [14]. These screens have uncovered an important principle of ASEL/R laterality through the identification of so-called Class I “2-ASEL” and Class II “2-ASER” mutants [15]. In Class I mutants, both neurons adopt ASEL fate (as assessed by expression of ASEL-specific *gcy* chemoreceptors), while losing ASER fate; in Class II mutants the opposite happens in that both neurons adopt ASER fate and lose ASEL fate [16]. The molecular basis for this bistability is a double-negative feedback loop of transcription factors and at least one miRNA, the above-mentioned *lsy-6* miRNA (Figure 1C; [16], [17]). Through genetic epistasis analysis, we have found the earliest trigger of asymmetry to be the expression of the *lsy-6* miRNA [16]. Yet, it is not known how its expression is restricted to ASEL. Our screen has uncovered a novel regulator of ASE asymmetry, the chromatin methlytransferase complex COMPASS. Through analysis of known and novel mutants in components of this complex we show that the COMPASS complex acts during embryogenesis, before the birth of the ASE neurons, to regulate *lsy-6* expression. As such, it provides a potential molecular link between the early Notch-signal, *tbx-37/38* expression and ASEL-specific *lsy-6* expression in the maturing ASE neurons.

Taken together, studying the development of the ASE neurons provides a window to understanding a host of questions relevant to neuronal differentiation – how are early lineage decisions made, how are symmetry and asymmetry imposed, how are individual neuronal cell fate decisions controlled? Previous genetic mutant screens that used ASE differentiation markers as a read-out have begun to address these questions, but these genetic screens have not yet been saturated [18]. We sought to further expand the list of genes required to build an ASE neuron. To facilitate the recovery of lethal genes and to avoid the problem of mutational hotspots, we have used a genome-wide RNAi screen to reach this goal. To our knowledge, this is the first genome-wide RNAi analysis of a single neuron cell fate decision.

## Results

### Establishing conditions for a genome-wide neuronal RNAi screen

**Figure 2:**
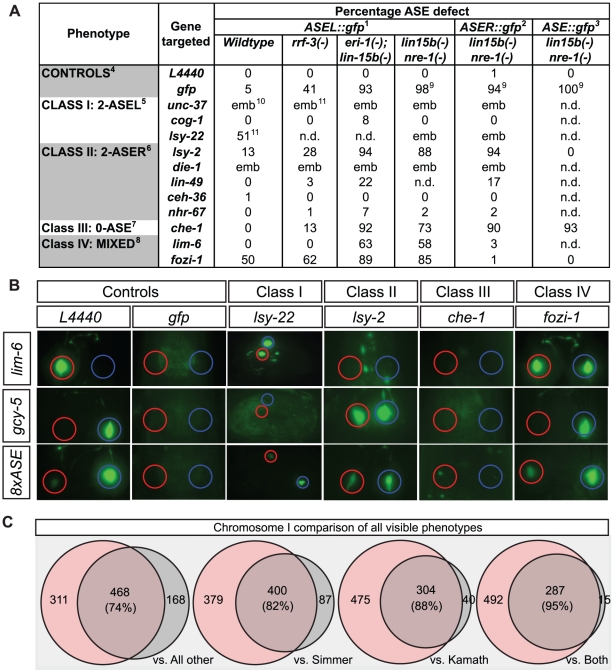
Testing hypersensitive strains and validating the screen with visible phenotypes. A: Table indicating the quantification of the laterality defects observed following RNAi knock-down of the indicated genes in wild-type (*otIs114*) and 3 different RNAi sensitized backgrounds. *nre-1(-) lin-15b(-)* and *eri-1(-); lin-15b* are the most sensitive strains for ASE. In all cases n>40. ^1^Scored with *otIs114*; ^2^Scored with *otIs186*; ^3^Scored with *otIs299*; ^4^ASE defect refers to loss or dimming of *ASEL::gfp, ASER::gfp* or *ASE::gfp*; ^5^ASE defect refers to ectopic expression of *ASEL::gfp* in ASER or loss or dimming of *ASER::gfp* or *ASE::gfp*; ^6^ASE defect refers to loss or dimming of *ASEL::gfp* or *ASE::gfp* and ectopic expression of *ASER::gfp* in ASEL; ^7^ASE defect refers to loss or strong dimming of *ASEL::gfp, ASER::gfp* or *ASE::gfp*; ^8^For *fozi-1* ASE defect refers to ectopic expression of *ASEL::gfp* in ASER or loss of *ASER::gfp* or *ASE::gfp*. For *lim-6* it refers to loss of *ASEL::gfp* expression or ectopic expression of *ASER::gfp* in ASEL*; ^9^ASEL::gfp* when still present appeared much dimmer than in any other strain; ^10^6/6 escapers displayed ectopic expression of *ASEL::gfp* in ASER; ^11^1/1 escaper displayed ectopic expression of *ASEL::gfp* in ASER; ^12^Taken from Flowers and Poole et al. 2010. B: Representative pictures of the ASE laterality that result from RNAi knock-down of the indicated known ASE factors in an *nre-1(-) lin-15b(-)* background. ASEL is labeled with *lim-6^prom^::gfp* and ASER is labeled with *gcy-5^prom^::gfp*. *L4440* refers to feeding with an empty RNAi vector. Examples of the 4 main classes of ASE phenotype as described in [18]. Class I is 2-ASEL, class II is 2-ASER, class III is 0-ASE (loss of ASE cell fate) and class IV is mixed (in the case of *fozi-1* ASEL markers are depressed in ASER but ASER markers are unaffected). C: Comparison of the visible phenotypes observed in our screen with previous screens for all genes on Chr I. Ovals represent the amount of bacterial clones that gave an RNAi phenotype in an experiment with results from our screen shaded in pink and previous published results shaded in grey. Areas that overlap represent clones (in %) for which in both experiments an RNAi phenotype was detected. Differences and overlap between an RNAi experiment done with the *lin-15b nre-1* mutant strain and (in the following order): (1) all data previously reported for an RNAi experiment on Chr. I (whether as part of a genome wide screen, subscreen or a single experiment, exported from wormbase.org WS190); (2) the data obtained by screen done with the hypersensitive *rrf-3* strain [22]; (3) the data obtained by screen done with the standard laboratory strain Bristol N2 [23]; (4) the data obtained by an overlap of phenotypes reported in both Simmer and Kamath screens.

RNAi is known to work less efficiently in the nervous system than in other tissues [19]. Indeed, we find that dsRNA directed against *gfp* barely reduces *gfp* expression levels in transgenic animals that drive a *gfp* reporter in the ASEL neuron (Figure 2A). Several genetic backgrounds have been described in which animals are more sensitive to RNAi. We tested three of these backgrounds, *nre-1 lin-15b* [20], *eri-1; lin-15b* [21] and *rrf-3* [22]. We find that in all of them ASE-specific *gfp* expression is now significantly reduced, with *nre-1 lin-15b* and *eri-1; lin-15b* mutants allowing more effective *gfp* knockdown than *rrf-3* (Figure 2A).

Previous mutant analysis from our lab has identified a number of genes required for the correct terminal differentiation of the ASE neuron (Figure 2A–2B; [18]). We tested whether RNAi recapitulates the mutant phenotype of 11 of these genes. We find that in a non-sensitized background, RNAi of only 3 of the genes partially recapitulates the mutant phenotype. In *rrf-3* mutants, the effects of knockdown improved for only some genes, but in both *nre-1 lin-15b* and *eri-1; lin-15b* mutants, RNAi phenocopies the effect of the respective mutant in most cases examined (Figure 2A–2B). Since *eri-; lin-15b* mutants generally seem less healthy than *nre-1 lin-15b* mutants, we chose the latter strain for our genome-wide screen.

As the *nre-1 lin-15b* mutant background is relatively untested for its overall sensitivity to RNAi in general, we screened all RNAi clones on chromosome I not just for ASE neuronal phenotypes (as we did for all chromosomes), but also for obviously visible phenotypes, (sterility, embryonic or larval lethality, slow post-embryonic growth, or post-embryonic morphological defects; see Materials and Methods) and compared those to previously reported chromosome I screens of visible phenotypes, conducted either in a wildtype or an *rrf-3* mutant background (Figure 2C; [22], [23]). We found a total of 779 clones with at least one visible phenotype detected, with an overlap of 82% and 88% for the *rrf-3* and wildtype Bristol N2 screens respectively. When compared to the group of clones for which phenotypes were reported in both *rrf-3* and wildtype N2 screens, our screen reveals 95% overlap. When we specifically compared the phenotypic categories reported by others and us, we found a striking trend of genes being shifted into more severe phenotypic categories in the *nre-1 lin-15b* background. For example, of the 303 clones reported previously to produce a Gro phenotype (slow growth), we found 57% to also be Gro in our screen but an additional 22% were found in more severe non-viable categories (Figure S1).

### A genome wide RNAi screen identifies 245 genes that affect expression of an ASEL-specific neuronal fate marker

**Figure 3:**
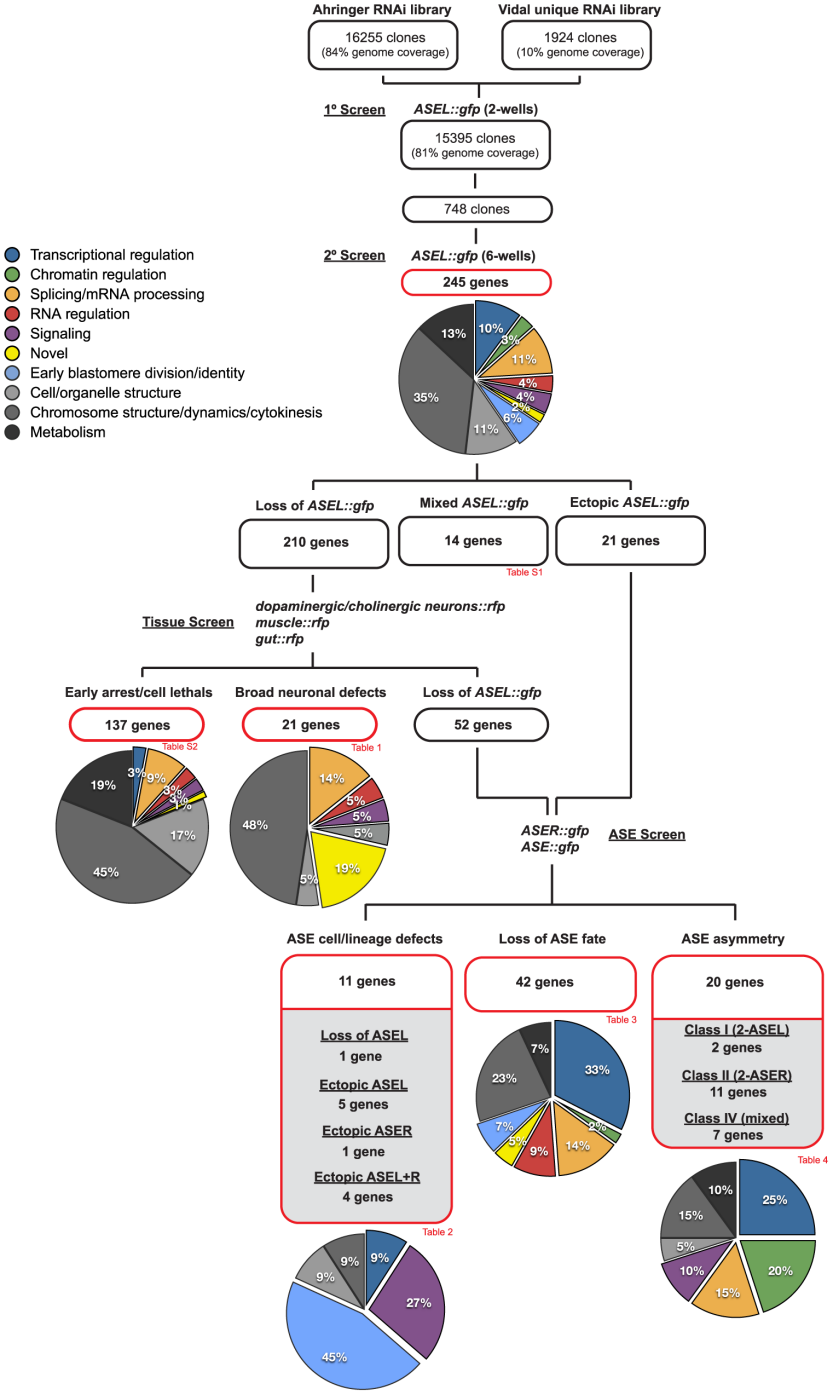
Overview of the screening strategy. A flow chart illustrating the screening strategy. The number of RNAi clones screened is indicated and at each step the number of genes identified in each phenotypic category is depicted. The pie-charts illustrate the percentage of genes in each of several defined functional categories at each step (see color key). See the text for details on categorization criteria and see the indicated tables (Table 1, Table 2, Table 3, Table 4, and Tables S1 and S2) for the list of genes in each category, a more detailed phenotypic description and their functional categorization.

**Figure 4:**
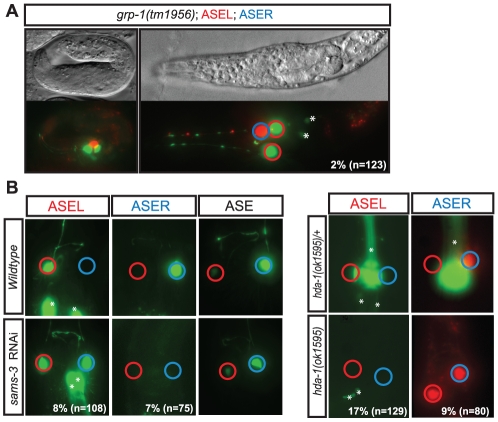
Examples of phenotypic classes uncovered by the screen. A: ASE cell/lineage defect—*grp-1(tm1956)* mutants, a GTP exchange factor, display ASE lineage defects in embryonic and larval stages. In the figure shown the animal has a total of 3 ASEs. One that displays ASER fate (blue circle), and instead of one that displays ASEL fate as seen in wild-type animals, *grp-1(-)* animals display two ASEL fated cells (red circles), suggesting an ectopic ASEL lineage has formed in these mutants. B: ASE asymmetry defects—Panel on the left shows RNAi knockdown of gene S-adenosylmethionine synthetase (*sams-3*) results in a class I “2-ASEL” phenotype in which ASER (blue circle) looses its sub-type specific fate and transforms to ASEL (red circle). Overall ASE fate is unaffected. We observed a similar phenotype with similar penetrance for *sams-4* (data not shown). Panel on the right shows the phenotype of histone deacetylase *hda-1(ok1595)* mutants which display a class II “2-ASER” defect. ASEL fate is reported by *lim-6^prom^::gfp* expression, ASER fate is reported by *gcy-5^prom^::gfp* or *gcy-5^prom^::mCherry* and ASE fate is reported by *8XASE::gfp*. *Indicates expression unrelated to the ASE neurons.

Using the *nre-1 lin-15b* sensitized background and the ASEL-marker *lim-6::gfp* (*otIs114* transgene), we then went on to conduct a primary screen of all available clones. We were able to screen 15,395 RNAi clones for ASEL defects, equaling 81% of genes in the genome. In the primary screen, all clones were scored in duplicate, revealing that 748 RNAi clones affect development of the ASEL neuron, as evidenced by loss or ectopic expression of the *lim-6::gfp* marker. These 748 clones were re-screened 6 additional times and we set the arbitrary threshold of considering any clone that repeated at least 3/8 times as positive. These criteria identified a total of 245 genes that affect the development of the ASEL neuron (Figure 3). Examples of some of the knockdown phenotypes are shown in Figure 4 (showing either the RNAi phenotype or mutant alleles that confirm the RNAi phenotype). The vast majority of these genes (237/245) cause embryonic lethality, in addition to the ASEL phenotype. Of these 245 genes, 21 genes resulted in ectopic expression of the ASEL marker and 210 genes resulted in a loss of ASEL marker expression when knocked-down. 14 genes displayed a mixed phenotype, i.e. individual animals within a population of treated animals show either no phenotype, loss or ectopic expression of the ASEL marker (this category was not further pursued; Table S1).

#### Early arrest

To determine whether loss of ASEL marker expression was due to (a) a specific loss of ASEL, (b) a more broad neuronal differentiation defect or (c) general early embryonic arrest, we re-screened the 210 genes that resulted in a loss of ASEL with fluorescently labeled differentiation markers for ectoderm, mesoderm or endoderm. We used two neuronal markers that label 6 different classes (each class composed of a pair of symmetric neurons) of dopaminergic and cholinergic neurons with limited lineage relation (*dat-1::rfp; ttx-3::rfp*, both contained on one array), a collagen marker for hypodermis (*dpy-7::yfp*), a myosin muscle marker (*myo-3::gfp*) and a gut-specific GATA factor marker (*elt-2::gfp*). We considered all those genes that by RNAi led to a loss of all tissue markers tested as “early arrest” genes (87 genes; Table S2). Additionally, we included in this list those genes that have previously been characterized as arresting before the 200-cell stage (11 genes; [24]) and those genes whose knock-down resulted in a loss of all markers tested except the dopaminergic neuron marker, as we presume this to result from non-reproducibility of RNAi (8 genes; we note that as expected, knock-down of these genes also led to a loss of expression of an ASER marker and a bilateral ASE marker). Finally, we also considered as early arrest those genes whose knock-down led to a loss of neurons and muscle but not gut, since the latter marker is expressed early in development before terminal neuronal differentiation (31 genes). In this manner we classified a total of 137 of those genes that led to a loss of *lim-6::gfp* expression as “early arrest” genes (Figure 3 and Table S2). More than 80% of genes in this category classify as genes involved in metabolism, cell/organelle structure and basic cellular processes like cell division.

#### Broad neuronal defects

Interestingly, we were also able to identify from this tissue screen 21 genes that when knocked-down by RNAi led to a broad loss of neurons (Figure 3 and Table 1). In all cases, in addition to a loss of the ASEL marker, the dopaminergic/cholinergic markers were absent, yet either muscle, or muscle and gut, were unaffected. We note that in all cases the expression of the hypodermal marker was absent, which is consistent with a loss of all ectodermal tissue. Genes in this category include two *mex* genes, known to be involved in determining the identity of the AB blastomere (which produces mainly ectodermal tissue), with their loss leading to excess P-cell descendants such as muscle, being generated ectopically from the AB-lineage (hence *m*uscle *ex*cess for *mex* [25]; Table 1). We suspect that other genes in this category are also involved in establishing early blastomere asymmetry and identity. We examined these and several other genes in this category in more detail by performing RNAi in the background of a pan-neuronally expressed gene (*rab-3::yfp*). While both *mex-3* and *mex-5* led to an almost complete loss of neurons, no other gene examined generated such a complete loss of neurons, suggesting they generate broad neuronal but not pan-neuronal differentiation defects (Figure S2).

**Table 1:** Genes that elicit broad neuronal defects when knocked-down by RNAi.

Gene Name	Functional Category	Domain/Function	ASEL	Neurons	Muscle	Gut	Hyp	ASER	ASE
*tbg-1*	cell/organelle	Tubulin	-	-	+		-		
*his-42*	chromosome	Histone H3	-	-	+	+	-		
*zyg-9*	chromosome	Microtubule-associated protein	-	-	+	+	-		
*R04F11.3*	chromosome	DNA polymerase subunit Cdc27	-	-	+	+	ne		
*R53.6*	chromosome	DNA replication (GINS complex subunit)	-	-	+	+	-		
*his-3*	chromosome	Histone H2A	-	-	+	+	-		
*csc-1*	chromosome	Subunit of the Aurora B kinase complex	-	-	+	+	-	-	-
*his-13*	chromosome	Histone H3	-	-	+	+	-		
*his-4*	chromosome	Histone 2B	-	-	+	-	-		
*pole-2*	chromosome	DNA polymerase epsilon, subunit B	-	-	+	-	-		
*ran-3*	chromosome	Spindle formation (RCC repeat)	-	-	+	-	-		
*gpr-1*	blastomere	G protein regulator (GoLoco motif)	-	-	+	+	-		
*mex-3*	blastomere	RNA binding KH domain	-	-	+	+	-	-	ne
*mex-5*	blastomere	RNA binding CCCH domain	-	-	+	+	-		
*gpr-2*	blastomere	G protein regulator (GoLoco motif)	-	-	+	-	-		
*T01H3.4*	novel	Transmembrane domains	-	-	+	+	-		
*C30B5.4*	RNA reg.	RNA binding RRM domain	-	-	+	-	-		
*T09A5.9*	signalling	Regulatory subunit protein phosphatase 1	-	-	+	+	-		
*F19F10.9*	splicing	SART-1 family (U4/U6.U5 snRNP associated protein)	-	-	+	+	-		
*M03C11.7*	splicing	Pre-mRNA processing factor 3	-	-	+		-		
*D1054.14*	splicing	PRP38-like splicing factor	-	-	+	-	-		

See Materials and Methods for a detailed description of scoring criteria. Abbreviations: ne, not embryonic lethal.

#### Neuron-type specific defects

We then re-screened the remaining genes, that show specific ASEL defects (52 genes with a loss of ASEL marker and 21 genes that showed ectopic ASEL marker expression), with an ASER specific marker and an ASE bilateral marker in order to distinguish between the following three RNAi knock-down phenotypic categories: (1) genes that cause ASE cell/lineage defects (loss or ectopic ASEL and/or ASER cells – but not a complete loss of ASE cell fate); (2) genes that cause bilateral loss of ASE cell fate; (3) genes that cause ASEL versus ASER asymmetry defects. Firstly, we classified those genes that showed ectopic expression of the ASEL marker in additional cells, with the ASER marker being unaffected (or vice versa) as having an ectopic ASEL (or ASER) lineage and we classified those genes that showed a loss of the ASEL marker with the ASER being unaffected (or vice versa) as having lost an ASEL (or ASER) lineage. These genes may alter the fate of entire lineage branches or they may cause individual cells to lose/acquire an ASE fate – importantly ASE fate in general is not lost. Altogether we identified 11 genes with ASE cell/lineage defects (Figure 3 and Table 2). Nearly half of the genes that fall into this class are genes that are previously described as affecting early blastomere identities, including, for example, *par* genes [26].

**Table 2:** Genes that elicit ASE cell/lineage defects when knocked-down by RNAi.

Gene Name	Functional Category	Domain/Function	ASEL	>2 ASEL	ASER	>2 ASER	ASE	>2 ASE	Viable	ASE lineage phenotype	Mutant phenotype
*lis-1*	cell/organelle	WD40	-		wt		+/−			Loss ASEL	
*lrg-1*	chromosome	*let-99* related (pseudogene)	+		+/−		+	+		ectopic ASEL^1^	
*par-3*	blastomere	PDZ domain	+		+	+	+	+		ectopic ASEL^1^	
*mei-2*	blastomere	Microtubule regulator (similar to katanin)	+		-		+/−	+		ectopic ASEL^2^	
*par-2*	blastomere	RING finger	+	+	-		ne			ectopic ASEL^2^	Yes
*lit-1*	blastomere	Serine/threonine kinase (Wnt signalling)	-		+	+	+	+		ectopic ASER	
*pie-1*	blastomere	Zinc finger, CCCH-type	+	+	-		+	+		ectopic ASEL^2^	
*ced-3*	signalling	Caspase	+	+	+		+	+	Yes	ectopic ASEL^1^	Yes
*oac-35*	signalling	Integral membrane O-acyltransferase	+	+	-		ne			ectopic ASEL^2^	
*wwp-1*	signalling	E3 ubiquitin ligase	+		+	+	+/−	+		ectopic ASEL^1^	
*ref-1*	transcription	bHLH	+		wt		+	+	Yes	ectopic ASEL	Yes

See Materials and Methods for a detailed description of scoring criteria. Abbreviations: ne, not embryonic lethal; wt, wildtype.

1 also ectopic ASER lineage.

2 also loss of ASER lineage.

Secondly, we classified those genes that result in a loss of both the ASEL and the ASER cell fate markers, as loss of ASE fate (as expected, knock-down of the majority of these genes also resulted in no expression of a bilateral ASE marker). In addition, one gene, although seemingly wildtype for the ASER marker did not express a bilateral ASE marker and was added to this group. A total of 42 genes fell into this group (Figure 3 and Table 3). Note that, as stated above, dopaminergic and cholinergic neuron markers are unaffected by these genes. A majority of genes in this list have a role in gene regulation, either on the DNA or RNA level. The list includes a known regulator of ASE specification, the ASE terminal selector *che-1* [15], [27], and the bHLH gene *hlh-14* that we describe in more detail later in this paper.

**Table 3:** Genes that elicit a loss of ASE fate when knocked-down by RNAi.

Gene Name	Functional Category	Domain/Function	ASEL	Neurons	Muscle	Gut	Hyp	ASER	ASE	Viable	Mutant phenotype
*hda-1*	chromatin	Histone deacetylase	-	+	-	+	-	-	-		Yes
*cdk-7*	chromosome	Cyclin-dependent Kinase	-	+	+	-	-	-	-		
*cdk-9*	chromosome	Cyclin-dependent Kinase	-	+	+	+	-	-	-		
*csnk-1*	chromosome	Casein Kinase	-	ne	+	ne	+	-	-		
*F09D1.1*	chromosome	Ubiquitin hydrolase	-	+	-	+	-	-	-		
*him-10*	chromosome	Nuf2 kinetochore homolog	-	+	-	+	-	-	+/−		
*rfc-1*	chromosome	Replication factor C	-		-	+	-	-	ne		
*rpb-8*	chromosome	RNA polymerase subunit 8	-	+	+	-	-	-	-		
*spdl-1*	chromosome	Coiled coil protein required for chrom segregation	-	+	+	+	+	-	ne		
*sur-6*	chromosome	Protein phosphatase 2A, regulatory subunit	-	+	-	+	-	-	+/−		
*aph-1*	blastomere	Transmembrane protein req. for Notch signaling	-	+	+	+	+	-	ne		
*par-6*	blastomere	PDZ domain	-	+	+	-	-	-	+/−		
*glp-1*	blastomere	Notch	-	+	+	+	-	-	-		Yes
*pfd-6*	metabolism	Prefoldin	-		-	+	-	-	+/−		
*rnf-113*	metabolism	Hydrolase (HAD) and RING finger	-	+	+	+	-	-	-		Yes
*mel-32*	metabolism	Serine hydroxymethyltransferase (Ser->Gly)	-	-	+	-	+	-	wt		
*ccdc-55*	novel	Conserved coiled-coil	-	+	+	+	-	-	-		
*F37C12.1*	novel	Conserved coiled-coil	-	+	+	-	-	-	-		
*mog-4*	RNA reg.	DEAH helicase	-	+	+	+	-	-	ne		No^1^
*mog-5*	RNA reg.	DEAH helicase	-	ne	+	-	-	-	-		No^1^
*rnp-6*	RNA reg.	RNA-binding RRM domain	-	+	+	+	-	-	+/−		
*mag-1*	RNA reg.	MAGonashi homolog	-	+	+	+	-	-	wt		No^1^
*D1043.1*	splicing	INTS7 homolog; Integrator complex component	-	+	-	-	-	+/−	+/−		
*F23F1.5*	splicing	Snurportin	-	+	+	+	+	-	+/−		
*K07C5.6*	splicing	Pre-mRNA splicing Prp180-interacting	-	+	+	-	-	-	-		
*nxt-1*	splicing	RNA export factor	-	+				-	ne		
*ZC376.6*	splicing	Integrator subunit	-	+	+	+	-	-	+/−		
*Y65B4A.6*	splicing	Tsl. Initiation factor (DEAD/DEAH helicase)	-	+				-	wt		
*che-1*	transcription	ZF - C2H2 - 4	-	+	+	+	+	-	-	Yes	Yes
*F28B3.1*	transcription	Disco (C2H2) -interacting protein AMP-binding	-	ne	+	-	-	-	ne		No^1^
*hlh-14*	transcription	bHLH	-	+		+	+	-	-	Yes	Yes
*let-49*	transcription	Mediator complex	-					-	ne		No^1^
*mdt-17*	transcription	Mediator complex	-	+				wt	-		
*pqn-38*	transcription	Mediator complex	-	+	+	+	-	-	-		No^1^
*sel-8*	transcription	Mastermind homolog in Notch signaling	-	+	+	+	-	-	-		
*skn-1*	transcription	bZIP	-	+	+		ne	-	-		
*sknr-1*	transcription	bZIP	-	+	+	+	-	-	-		
*F54D5.11*	transcription	TFIIE beta subunit	-	+	+	-	-	-	-		
*taf-1*	transcription	TBP-associated factor (bromodomain)	-	ne	+	+	-	-	-		No^1^
*taf-5*	transcription	TBP-associated factor (WD40 domain)	-	+	+	+	-	-	-		
*tlf-1*	transcription	TATA binding TFIID	-	+	-	-	-	-	+/−		Yes
*let-526*	transcription	ARID/BRIGHT	-	+	+	+	+	-	wt		No^1^

See Materials and Methods for a detailed description of scoring criteria. Abbreviations: ne, not embryonic lethal; wt, wildtype.

1 possible reasons that the mutant phenotype did not recapitulate the RNAi phenotype include maternal rescue (of balanced lethals), non-null alleles, off-target effects of the RNAi clone or synergy with the *nre-1(-) lin-15b(-)* background.

#### L/R asymmetry defects

Finally, we identified 20 genes, knock-down of which caused an ASE asymmetry defect (Figure 3 and Table 4). We classified genes as having an asymmetry defect when the bilateral ASE marker is unaffected but ectopic expression of either the ASEL or ASER marker is observed. In addition, 4 genes for which the result from the bilateral marker was ambiguous, but which clearly showed loss of the ASEL marker and expression of the ASER marker in one extra cell or vice versa were added to this list. Ten genes showed ectopic expression of the ASEL marker, in 2/10 the ASER marker was lost, indicating a Class I (2-ASEL) phenotype, while in 7/10 the ASER is unaffected indicating a Class IV (mixed) phenotype (see Figure 2B). 11 genes showed a Class II (2-ASER) phenotype. This list includes a number of previously described regulators of ASE laterality, such as *die-1*, *lsy-22*, *lsy-2*, *fozi-1* and *lim-6* and, in addition, some novel genes that we describe later on in this paper.

**Table 4:** Genes that elicit ASE asymmetry defects when knocked-down by RNAi.

Gene Name	Functional Category	Domain/Function	ASEL	ASER	ASE	Viable	RNAi ASE phenotype	Mutant phenotype
*klp-16*	cell/organelle	Kinesin-like	+	wt	wt		Class IV (mixed)	Yes
*F21H12.1*	chromatin	Histone methyltransferase complex (WD40 domain)	+	+	wt	Yes	Class II (2-ASER)^1^	Yes
*lin-49*	chromatin	Histone acetylltransferase complex (bromodomain)	-	+	wt	Yes	Class II (2-ASER)	Yes
*ash-2*	chromatin	Histone methyltransferase complex (SPRY domain)	+	+	wt		Class II (2-ASER)^1^	Yes
*dpy-30*	chromatin	Histone methyltransferase complex	+	+	wt		Class II (2-ASER)^1^	Yes
*uri-1*	chromosome	Prefoldin	+	-	ne		Class I (2-ASEL)	
*smk-1*	chromosome	Regulatory subunit of the protein phosphatase 4	-	+/−	+/−		Class II (2-ASER)	
*tac-1*	chromosome	Microtubule-based processes (TACC protein family)	+	wt	wt		Class IV (mixed)	
*sams-3*	metabolism	S-adenosylmethionine synthetase	+	-	wt	Yes	Class IV (mixed)	
*sams-4*	metabolism	S-adenosylmethionine synthetase	+	-	wt	Yes	Class IV (mixed)	
*grp-1*	signaling	Guanine nucleotide exchange factor	+	wt	wt	Yes	Class IV (mixed)	Yes
*smo-1*	signaling	SUMO	-	+	wt		Class II (2-ASER)	Yes
*C07A9.2*	splicing	G10 splicing factor family (BUD31 ortholog)	+	wt	wt		Class IV (mixed)	
*rsp-7*	splicing	SR protein family (RNA binding RRM domain)	-	+/−	+/−		Class II (2-ASER)	
*ddx-23*	splicing	U5 snRNP-like RNA helicase subunit (DEAD and helicase)	-	+	wt		Class II (2-ASER)	
*die-1*	transcription	C2H2 Zn finger transcription factor	-	+	wt		Class II (2-ASER)	Yes
*lsy-22*	transcription	Groucho-like	+	-	+/−		Class I (2-ASEL)	Yes
*lsy-2*	transcription	C2H2 Zn finger transcription factor	-	+	wt	Yes	Class II (2-ASER)	Yes
*fozi-1*	transcription	C2H2 Zn finger transcription factor	+	wt	wt		Class IV (mixed)	Yes
*lim-6*	transcription	LIM homeobox	-	+	wt	Yes	Class II (2-ASER)	Yes

See Materials and Methods for a detailed description of scoring criteria. Abbreviations: ne, not embryonic lethal; wt, wildtype.

1 a low penetrant Class I (2-ASEL) phenotype was also observed.

Taken together, our RNAi screen uncovered a rich set of genes that affect early development, broad neuronal development, lineage specification and terminal ASE differentiation. Functionally, genes fall into a variety of different categories, from signaling to gene regulatory factors. In the second and third parts of this paper we undertake a more in-depth analysis of two sets of genes retrieved from our screen: (1) One specific transcription factor, the bHLH transcription factor *hlh-14*; (2) several genes that code for members of a specific histone-methylation complex, called COMPASS.

### The Achaete-Scute-like bHLH gene *hlh-14* controls neuronal fate induction

**Figure 5:**
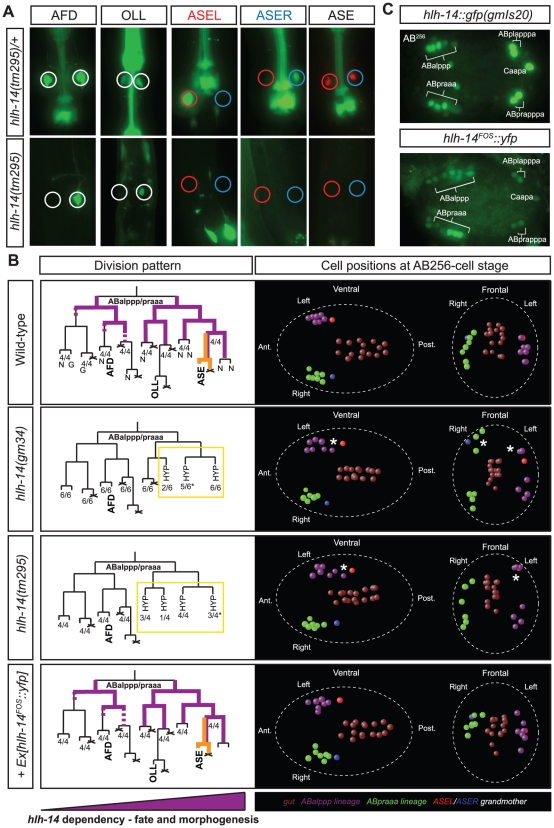
The *achaete-scute* homolog *hlh-14* is required to specify neurons in the ASE lineage. A: *hlh-14* is required for the terminal specification of several neuron pairs from the ABalppp/praaa lineage branch. Images of *hlh-14(tm295)* mutants indicating a loss of ASE (ASEL: *lim-6^prom^::gfp*, ASER: *gcy-5^prom^::gfp* and ASEL/ASER: *che-1^prom^::rfp*) OLL (*ser-2^prom2^:gfp*) and AFD (*gcy-8::gfp*) cell fates. Heterozygous balanced siblings are unaffected (presence of balancer indicated by GFP in the pharynx). B: Lineage, 3D-morphogenesis and *hlh-14* expression analysis in the ABalppp/praaa lineage of wild-type (containing the *ntIs1* transgene), *hlh-14(gm34); otIs114*, *hlh-14(tm295); ntIs1* and *hlh-14(tm295); otEx4507*, Ex*[hlh-14^FOS^::yfp]; ntIs1* embryos. The left panel shows the division pattern and expression (purple lines) of *hlh-14* in the ABalppp/praaa lineage as determined by 4D-lineage analysis. *hlh-14* expression was assessed in both *gmIs20* and *hlh-14(tm295); Ex[hlh-14^FOS^::yfp]* animals. In wild-type embryos the cells divided as expected. In *hlh-14(gm34)* mutants the 3 most posterior great-granddaughters of the ABalppp/praaa blastomere fail to divide. In *hlh-14(tm295)* mutants these defects extend to the 4 most posterior granddaughter. The number of lineages that divided as indicated by the lineage and the total number of individual left or right lineages followed to terminal division (and assessed for 3D cell positions) are shown at the bottom of each lineage diagram. The asterisks indicate that on one occasion the lineages still divided but aberrantly, much later than normal. N indicates neurons and G indicates glia. The right panel shows representative examples of the 3D-positions of cells from the ABalppp/ABpraaa lineage at the AB^256^-cell stage as assessed by 4D-lineage analysis. Both ventral and frontal views are presented. The posterior descendants of the ABalppp/praaa blastomere are dorsally and posteriorly mispositioned in *hlh-14* mutants (asterisks), a position normally occupied by hypodermal cells. The fosmid array rescues the division and morphogenesis defects. The gut lineage is indicated for positional information. Cell coloring, except C-lineage, after [10]. The purple triangle under the panel indicates that *hlh-14* expression is lost more rapidly in the anterior of the lineage and this correlates with more penetrant defects in posterior parts of lineage in *hlh-14* mutants. C: Images of *hlh-14* expression in embryos at the pre-morphogenesis (AB^256^-cell) stage. Top panel shows the expression of *hlh-14* from an integrated translational *hlh-14::gfp* reporter [28]. Bottom panel shows the expression of *hlh-14* from a rescuing YFP-tagged *hlh-14* fosmid array. Expression in the ASE, PVQ/HSN/PHB, and DVC/PVR lineages is indicated. The full expression pattern of *hlh-14* in the ABalppp/praaa lineage is indicated by purple lines in the lineage diagram in panel C and full expression in the C-lineage is indicated in Figure 6.

The RNAi screen identified the *hlh-14* gene as affecting overall differentiation of ASEL/R. *hlh-14* RNAi animals are viable and display a loss of asymmetrically expressed and bilaterally expressed genes in ASE to varying extents (Table 5). We first set out to corroborate the RNAi phenotype using available mutant alleles of *hlh-14*. We found that two different alleles of *hlh-14* recapitulated the ASE differentiation phenotype observed upon RNAi and were larval lethal as previously reported [28]. Asymmetrically expressed genes are lost in *hlh-14* mutants, as is the expression of *che-1*, a terminal selector of ASE neuron differentiation (Figure 5A and Table 5). The defects are almost completely penetrant in *hlh-14(tm295)*, a deletion allele that removes the entire bHLH domain and is likely a functional null.

**Table 5:** *hlh-14* is required for the ASE neurons and several other neurons in the same lineage branch.

	Phenotype		
Genotype	Loss	Partial loss^1^	Intact
*hlh-14(RNAi); nre-1(hd20) lin-15b(hd126)*	50%	6%	100%
*hlh-14(tm295)/+*	0%	0%	100%
*hlh-14(tm295)*	97%	0%	3%
*hlh-14(gm34)/+*	0%	0%	100%
*hlh-14(gm34)*	77%	0%	23%
*hlh-14 (RNAi); nre-1(hd20) lin-15b(hd126)*	7%	1%	92%
*hlh-14(tm295)/+*	0%	0%	100%
*hlh-14(tm295)*	99%	0%	1%
*hlh-14(gm34)/+*	0%	0%	100%
*hlh-14(gm34)*	88%	0%	12%
*hlh-14(tm295) + Ex[hlh-14^FOS^::gfp] #1*	5%	0%	95%
*hlh-14(tm295) + Ex[hlh-14^FOS^::gfp] #2*	4%	0%	96%
*hlh-14(tm295) + Ex[hlh-14^FOS^::gfp] #3*	10%	0%	90%
*hlh-14(tm295) + Ex[hlh-14^FOS^::gfp] #4*	2%	0%	98%
*hlh-14 RNAi; nre-1(hd20) lin-15b(hd126)*	4%	26%	70%
*hlh-14(tm295)/+*	0%	3%	97%
*hlh-14(tm295)*	95%	5%	0%
*hlh-14(tm295)/+*	0%	0%	100%
*hlh-14(tm295)*	60%	38%	2%
*hlh-14(tm295)/+*	0%	0%	100%
*hlh-14(tm295)*	6%	29%	65%

Phenotypic characterization and rescue of *hlh-14* mutants and *hlh-14* RNAi. In all cases n>40.

1 Partial loss refers to a diming in the case of those markers that are asymmetrically expressed and a loss of one cell for those makers bilaterally expressed.

2 Scored with *lim-6::gfp(otIs114).*

3 Scored with *gcy-5::gfp(otIs186)*; See Materials and Methods for fosmid array details.

4 Scored with *che-1::gfp(otIs217)* except for the RNAi which was scored with *8xASE::gfp(otIs299)*.

5 Scored with *ser-2^prom3^::gfp(otIs138)* or *8xASE::gfp(otIs299)*.

6 Scored with *gcy-8::gfp(oyIs17)*.

We find that other neurons that are lineally related to ASE are also affected in *hlh-14* mutants, as assessed by loss of terminal fate marker expression of the OLL and AFD neuron pairs. These broader neuronal defects were not apparent during initial RNAi screening since the dopaminergic and cholinergic neuron marker was unaffected and *hlh-14(RNAi)* animals are viable, unlike *hlh-14* mutants which are larval lethal. Intriguingly, the effect on fate appears to be more pronounced the more posterior a neuron is located in the lineage (Figure 5A and Table 5).

We analyzed the *hlh-14* mutant phenotype in more detail using a classic lineaging approach, in which we monitored cell position, cleavage and morphology, using 4D microscopy [10]. We find that from the AB^64^-cell stage onwards, during gastrulation, the ABalpppp/ABpraaap neuroblasts and their descendants migrate dorsally, towards a location usually populated by hypodermal cells [6], rather than remaining more ventrally positioned as they do in wild-type animals. By the AB^256^-cell stage these descendants are both dorsally and posteriorly mislocalised (Figure 5B). At this stage, the time at which hypodermal cells normally cease division and differentiate, these descendants fail to divide further, unlike wild-type cells, which undergo one or two more rounds of division before differentiating into neurons. Furthermore, in certain cases where we were able to follow the cells for a sufficient period of time, we observed that they acquired a hypodermal-like cell morphology. We observe more frequent hypodermal transformations and mis-positioning defects in more posterior descendants of the ABalpppp/ABpraaap neuroblasts (Figure 5B). Additionally, we examined the hypodermal cell fate marker *dpy-7* in *hlh-14*(*tm295*) mutants at the comma stage (Figure S3). Although we were able to easily observe additional cells in the posterior of the embryo, consistent with previously reported cell fate transformations [28] and novel tranformations that we describe below, we were unable to unambiguously identify extra hypodermal cells in the anterior of the embryo due to the dim expression of *dpy-7* in certain anterior hypodermal cells (we note that several anterior hypodermal cells, in addition to the hypodermal seam cells, do not appear to express *dpy-7* at this stage). Nevertheless we were able to observe mispositioned hypodermal cells in the anterior of the *hlh-14(tm295)* mutants, where the transformed cells of the ABalpppp/ABpraaap reside (Figure S3). The division and dorsal mispositioning defects are more severe in the null allele of *hlh-14* (Figure 5B). Together, these data are consistent with a neuron-to-hypodermal transformation of these cells and suggest that, in addition to cell fate, *hlh-14* regulates aspects of cell migration.

To examine *hlh-14* gene expression, we generated a YFP-tagged *hlh-14* transgene through fosmid recombineering (Figure 5C; [29]). This fosmid reporter fully rescues the lethality, division, morphogenesis and ASE cell fate defects of *hlh-14* mutants (Figure 5B and Table 5). We find, using 4D-lineage analysis, that this reporter and a previously published *hlh-14* translational reporter [28] show similar expression in the ASE lineage. *hlh-14* is first expressed at the AB^64^-cell stage (Figure 5B). This is just shortly after the point at which the ASE-lineages, which descend from distinct asymmetric blastomeres, become symmetric in terms of division pattern and daughter cell fates; the descendants will form exclusively neurons and glia, producing the left-right bilateral homologs of eleven pairs of neurons and two pairs of glia (Figure 1). It is also the time we begin to see morphogenesis defects and together is consistent with a role of *hlh-14* being a bilateral binary switch at, or shortly after the ABalpppp/ABpraaap stage, to determine neuronal versus hypodermal fate.

*hlh-14* expression appears to persist longer in posterior parts of the ABalppp/praaa lineage than in anterior parts, thereby mirroring the differential phenotypic severity of *hlh-14* removal (Figure 5A–5B). In the ASE branch, located at the posterior part of the lineage, *hlh-14* expression is maintained throughout the lineage until shortly before the terminal division of the ASE neurons, suggesting not only early neuronal versus hypodermal decision roles for *hlh-14* but perhaps also a later role in differentiation. Consistent with such a notion we find that on examining ASEL, partial removal of *hlh-14*, through RNAi in animals that express two distinct markers for ASEL fate simultaneously, sometimes leads to loss of one but not the other marker (13/28 embryos in which ASEL was still present, as assessed by a *lsy-6::yfp* reporter, lost *ceh-36::mCherry* expression) suggesting the ASE cell is still produced but fails to differentiate correctly.

### Symmetric and asymmetric expression of *hlh-14* in other neuronal lineages

**Figure 6:**
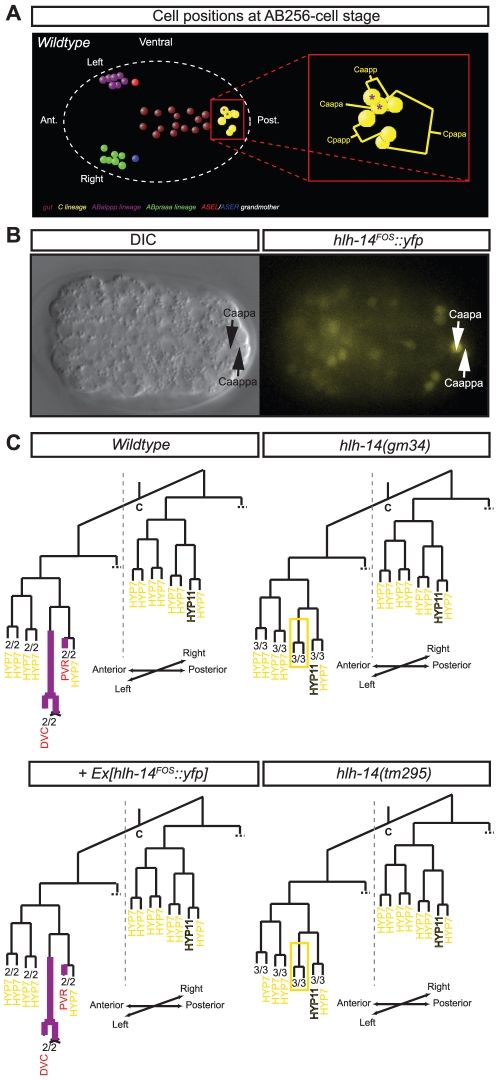
Asymmetric expression and function of *hlh-14* in the C-lineage. A: Ventral view of the 3D-positions of certain cells of the C-lineage of a representative wild-type embryo at the AB^256^-cell stage (shortly after the division of the ASE grandmothers), as assessed by 4D-lineage analysis. The left-sided asymmetric neuroblast Caapa and the neuron PVR express *hlh-14* (purple asterisks). Cells of the gut and the ASE lineages are shown for positional information. Cell coloring, except C-lineage, after [10]. B: Ventral view in both DIC and fluorescence of a representative lineaged *hlh-14(tm295*); Ex[*hlh-14^FOS^::yfp*]; *ntIs1* embryo at the AB^256^-cell stage. C: Panel shows the expression (purple lines) of *hlh-14* and the division pattern of part of the C-lineage as determined by 4D-lineage analysis of wild-type (containing the *ntIs1* transgene, *hlh-14(gm34); otIs114*, *hlh-14(tm295); ntIs1* and *hlh-14(tm295); otEx4507*, Ex*[hlh-14^FOS^::yfp]; ntIs1* embryos. *hlh-14* expression was assessed in both *gmIs20* and *hlh-14(tm295); Ex[hlh-14^FOS^::yfp]* animals. The Caapa blastomere divides early in *hlh-14* mutants, at a time when closely related cells produce hypodermal cells. This defect is rescued by the *hlh-14^FOS^::yfp* transgene. The extra hyp11 cell that can be observed expressing *dpy-7* (Figure S3), is indicated. The number of lineages that divided as indicated and the total number of individual left or right lineages followed to terminal division are shown at the bottom of each lineage diagram. Red cell names indicate neurons/glia and yellow cell names indicate hypodermis.

In addition to its expression in the ASE lineage, we observe by 4D-lineage analysis that our *hlh-14* fosmid reporter is expressed in several other neuronal lineages. In some instances this expression is bilaterally symmetric, such as in the neuroblasts that give rise to the L/R bilaterally symmetric PLM/ALN neuronal pairs, PVQ/HSN/PHB neuron pairs and the ALM/BDU neuron pairs (Figure S4). Surprisingly, in other instances this expression is L/R asymmetric, such as in neuroblasts of the C lineage (Figure 6). We observe asymmetric expression in two asymmetrically generated neuroblasts, the Caapa neuroblast that gives rise to the DVC neuron and a cell death, and the Caappv cell, which differentiates into the neuron PVR. The bilateral homologs of these *hlh-14-*expressing neuroblasts are non-neuronal hypodermal cells (Figure 6C). In both *hlh-14(gm34)* and *hlh-14(tm295)* mutants we observe that the Caapa neuroblast divides precociously, indicative of a hypodermal transformation. In the position of Caapaa, we observe an ectopic cell expressing the *dpy-7* hypodermal cell marker, corroborating that PVR has been transformed into *hyp11*, the normal fate of Caappv's bilateral homolog (Figure 6C). These division defects are rescued by the *hlh-14^FOS^::yfp* transgene (Figure 6C). Several other lineages in which we observe *hlh-14* expression also give rise mainly to hypodermal cells, the P-cells in the case of the PLM/ALN and PVQ/HSN/PHB lineages (Figure S4B) and the V-cells in the case of the ALM/BDU lineage (Figure S4C). In the PVQ/HSN/PHB lineage we also observe precocious division of the neuroblast that gives rise to these neurons and ectopic *dpy-7* expressing cells in the tail in the absence of *hlh-14* (Figure S3; data not shown). We suggest that *hlh-14* acts as a neural/hypodermal switch in the neuroblasts of all these lineages.

Intriguingly, we also observe expression in the bilaterally symmetric neuroblasts that produce a pair of pharyngeal interneurons known as I1L/I1R (Figure S5). The lineages that generate these neurons are unusual for two reasons: (1) they derive from L/R asymmetric origins and (2) they are multi-potent, giving rise to mesodermal, epithelial and neuronal cells of the pharynx. We find that in *hlh-14(tm295)* mutants the division of this neuroblast is again premature, more closely matching the division time of its sister, which does not express *hlh-*14 and normally gives rise to two myoepithelial cells, a muscle and a marginal cell (Figure S5B). While it is currently unclear what fate these cells acquire in the absence of *hlh-14*, it suggests that in addition to regulating neural/hypodermal fate decisions, *hlh-14* regulates more general neural/non-neural cell fate decisions and that “non-clonally” derived neurons use the same basic neural specification mechanisms as clonally derived neurons.

Finally, we observe later expression of *hlh-14* in currently unidentified lineages, at a time in which all terminally differentiated cells present at hatching are already formed. This is consistent with *hlh-14* acting during terminal cell fate decisions in other lineages. Taken together, *hlh-14* acts as a proneural gene to regulate early neuroblast cell fate, morphogenesis and certain aspects of terminal differentiation in multiple L/R symmetric and L/R asymmetric neuronal lineages. In the ASE lineage, its proneural activity is graded in an anterior-to-posterior manner.

### The COMPASS histone methyltransferase complex regulates ASE laterality

**Figure 7:**
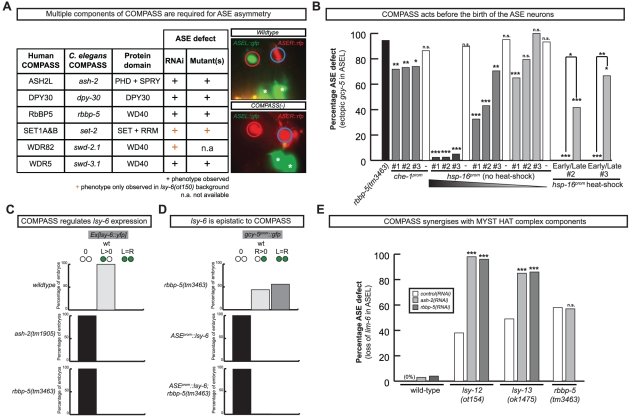
The evolutionary conserved methyltransferase COMPASS complex is required for ASE asymmetric cell fate specification. A: Table and representative images of COMPASS complex indicating the ASE phenotype in multiple components of the COMPASS complex (for detailed numbers see Figure S3). The image shown on the right is of the *lsy-15(tm3463)* mutant; all alleles of several members of the COMPASS complex show an identical phenotype. The strain contains a reporter that marks ASEL (*lim-6^prom^::gfp*) and a reporter that marks ASER (*gcy-5^prom^::mcherry*). The phenotype displayed is loss of ASEL (red circle) fate and a concomitant gain of ASER (blue circle) fate in ASEL. Asterisks denote expression unrelated to ASE. B: Rescue of the *rbbp-5* mutant phenotype with heterologous promoter lines (see Materials and Methods). ‘Early’ heat-shocks were performed on embryos younger than the AB^256^-cell stage, before the birth of ASE and ‘late’ heat-shocks on embryos older than the AB^256^-cell stage, after the birth of ASE. The *che-1::rbbp-5::gfp* construct was injected at 20 ng/ul and the *hsp-16::rbbp-5::gfp* construct was injected at 50, 20 and 2 ng/ul. Three lines of each were tested. Statistical significance (in comparison to the mutant alone unless otherwise indicated) was assessed using a z-test and a Bonferroni correction was applied for multiple comparisons (*p<0.05, **p<0.01, ***p<0.001). The ASE phenotype was scored with ASER marker *gcy-5^prom^::gfp (otIs220)*. In all cases n>30 except for the staged heat-shocks where n>8. C: Graphs indicating the left-right ASE expression of *lsy-6* in wild-type and COMPASS complex mutants. Ex[*lsy-6::yfp*] is an extrachromosomal array, *otEx4410* Ex[*lsy-6::yfp; ttx-3::mcherry*]. Only array carrying worms were scored. In all cases n>50, see Table S3 for more detailed numbers. D: Graphs indicating the left-right ASE expression of *gcy-5^prom^::gfp (ntIs1)* in *lsy-6* overexpressing, *rbbp-5(tm3463)* and double-mutant animals. Similar results were obtained with *rbbp-5(ot86)* (Table S3). In all cases n>50. *ASE::lsy-6* is *ceh-36^prom^::lsy-6 (otIs204)*. E: Graph indicating the percentage ASE defect following *control* (empty RNAi vector), *ash-2* and *rbbp-5* RNAi in wild-type, *lsy-12(ot154)*, *lsy-13(ok1475)* and *rbbp-5(tm3463)* mutant animals. The ASE defect was scored with the ASEL marker *lim-6^prom^::gfp (otIs114)*. Statistical significance (in comparison to control RNAi in relevant mutant background unless otherwise indicated) was assessed using a z-test (*p<0.05, **p<0.01, ***p<0.001). In all cases n>70. Numbers for control (empty vector) RNAi are taken from Figure 1. For more detailed numbers see Table S3. For the ASEL scoring of *ash-2* and *rbbp-5* RNAi in a wild-type background, RNAi was performed in both a strain containing either the *lim-6::gfp(otIs114)* reporter to match the reporter present in *lsy-12(ot154)* strain and in a strain containing both the *lim-6::gfp(otIs114)* and *gcy-5::rfp(otIs220)* reporter to match the *lsy-13(ok1417)* strain. No difference was observed and the numbers were pooled.

**Figure 8:**
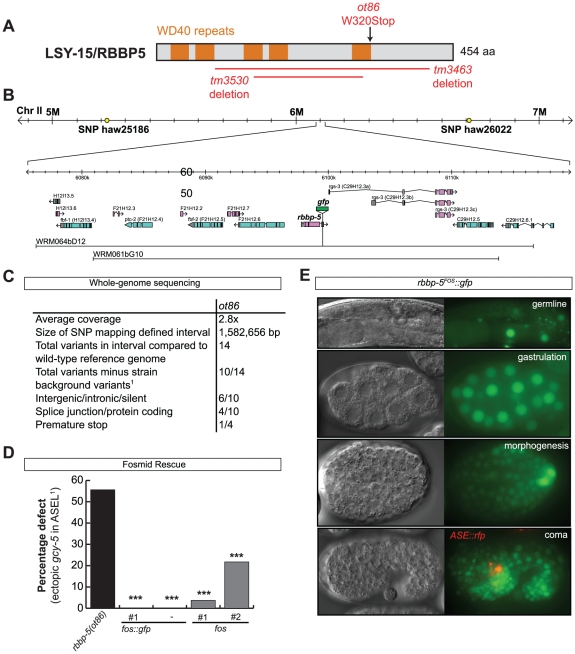
The cloning and expression of *lsy-15/rbbp-5(ot86)*. A: Protein schematic of LSY-15/RBBP-5 with the location and nature of mutant alleles identified and/or used in this study. B: Schematic illustrating the SNP-mapping and genomic region of *lsy-15/rbbp-5*, and the fosmids used to for expression and rescue analysis. *lsy-15(ot86)* was mapped to a ∼15 MB interval on Chr II between the polymorphisms *haw25186* and *haw26022*. Two different fosmids were used to rescue *lsy-15/rbbp-5(ot86)* mutants, one of which was recombineered to express a translational *lsy-15/rbbp-5::gfp* fusion for expression analysis. C: Whole genome sequencing (WGS) data for the *lsy-15(ot86)* allele. Only 4 protein coding variants resided in the interval to which *lsy-15(ot86)* had been mapped, one of which was a premature stop in the *rbbp-5* locus which was confirmed by Sanger sequencing. ^1^Variants were considered as background if they were also found in other WGS datasets from the lab (see Material and Methods). D: Rescue of the *rbbp-5* mutant phenotype with fosmid lines (see Materials and Methods). Statistical significance (in comparison to the mutant alone) was assessed using a z-test and a Bonferroni correction was applied for multiple comparisons (*p<0.05, **p<0.01, ***p<0.001). E: Representative images of the expression of the *rbbp-5^FOS^::gfp* transgene. The top three images are in a *lsy-15/rbbp-5(ot86)* background and the bottom image is in a *che-1^prom^::mcherry* background that is expressed specifically in ASE neurons. Overlapping expression can be observed.

Our RNAi screen revealed ASE laterality defects upon knockdown of two different components of the histone methyltransferase complex COMPASS [30], *ash-2* (a PHD/SPRY domain protein) and *dpy-30*. A third locus, *F21H12.1*, also generated ASE laterality defects and we find that this locus corresponds to the worm ortholog of an additional member of the COMPASS complex, the WD40 protein RbBP5, here-on referred to as *rbbp-5* (Table 4 and Table S3; Figure 7). We confirmed the *ash-2(RNAi)* and *dpy-30(RNAi)* ASE laterality defects observed during our RNAi screen using mutant alleles of the respective genes (Figure 7 and Table S3). In regard to *rbbp-5*, we noted that it resides in the mapping interval of the previously identified but uncloned *lsy-15* gene, an ASE laterality mutant retrieved from a large-scale mutagenesis screen [18]. As previously described, *lsy-15(ot86)* mutants show a conversion of ASEL to ASER fate [18]; this phenotype, like those of the other COMPASS members, is maternally rescued (see below). Additionally, *lsy-15(ot86)* mutants, like other COMPASS complex mutants, display a partially penetrant dumpy phenotype, a low brood size and an egg-laying defect (data not shown). The latter is likely the result of a uterine attachment defect (Cog phenotype) that can be observed in *lsy-15(ot86)* mutants along with ectopic expression of the *ceh-2* vulval cell marker (data not shown). Through whole genome sequencing, rescue, and analysis of deletion alleles provided by the *C. elegans* knockout consortia, we find that *lsy-15* indeed corresponds to *rbbp-5* (Figure 8, Table S3 and see below).

We analyzed several other members of the COMPASS complex that were not revealed by our RNAi screen, using RNAi, mutant alleles or both. We identified a SET-type methyltransferase component, *set-2* (a SET1A&B ortholog) and another two WD40 proteins, *swd-3.1* and *swd-2.2* (the WDR5 and WDR82 orthologs) as having ASE laterality defects similar to those observed in *rbbp-5*, *dpy-30* and *ash-2* mutants (Figure 7 and Table S3). The ASE laterality defects of *set-2* and *swd-2* were only revealed in a genetically sensitized *lsy-6(ot150*) background. Additionally, this background enhanced the penetrance of the RNAi defects observed in several other COMPASS complex components (Table S3). Together we find that all core components of the COMPASS complex regulate ASE laterality.

### The COMPASS complex acts during embryogenesis and upstream of the miRNA *lsy-6* to regulate ASE laterality

To determine when and where COMPASS acts to regulate ASE asymmetry we focused on *rbbp-5* and generated several reporter gene and rescue constructs. A GFP-tagged fosmid array that fully rescues the ASE laterality defects of *rbbp-5(ot86)* mutants is expressed ubiquitously during early embryogenesis and is predominantly nuclear localized (Figure 8D–8E). We observe similar expression of a translational reporter gene construct containing the intergenic region upstream of *rbbp-5* (data not shown). This is consistent with previously reported broad expression of several other components of the COMPASS complex [31], [32]. At later stages, during and after the birth of the ASE neurons, *rbbp-5* expression is significantly reduced throughout the embryo. We are able to observe overlapping expression with a reporter gene expressed bilaterally in the ASE neurons, however, we note that expression of the transgenic array in the ASE neurons was relatively infrequent despite the fact that array carrying animals are fully rescued (Figure 8D–8E). Additionally, we also observe complete rescue of the ASE laterality defects in non-array carrying worms derived from array positive mothers suggesting that the fosmid array can maternally rescue *rbbp-5(ot86)* mutants. Consistent with this we observe maternal germline expression of the *rbbp-5* fosmid (Figure 8E) and the mutants themselves are maternally rescued (data not shown). These observations provide a hint that *rbbp-5* and the COMPASS complex may act early during embryogenesis to regulate ASE asymmetry. To examine this possibility in more detail, we attempted to rescue the *rbbp-5* mutant phenotype by driving an *rbbp-5::gfp* translational fusion using two heterologous promoters: (1) the *che-1* promoter, which is exclusively expressed bilaterally in the mother of the ASE neurons and then in the mature ASE neurons throughout the life of the animal or (2) the ubiquitous *hsp-16* heat-shock promoter. We find that ASE-specific expression of *rbbp-5* only minimally rescues the *rbbp-5(tm3463)* mutant phenotype, despite being strongly expressed in the ASE neurons as expected (Figure 7B and Figure S6). In contrast, leaky expression from lines carrying the *hsp-16* construct and maintained at room temperature strongly rescues the ASE laterality defect in a dose dependent manner, despite rare expression in the post-mitotic ASE neurons (Figure 7B and Figure S6). We next performed staged heat-shocks using those lines with minimal rescue ability of the *rbbp-5* laterality defect at room temperature. We find that when the heat-shock is performed early during embryogenesis, before the birth of the ASE neurons, the ASE laterality defect is fully rescued (Figure 7B). In contrast, if the heat-shock is performed at or after the birth of the ASE neurons the defect is only weakly rescued. We conclude that *rbbp-5* acts before the generation of the ASE neurons.

The most upstream component of the previously described network of laterality factors is the ASEL-expressed miRNA *lsy-6*. We find that *lsy-6* expression is lost upon abrogation of various COMPASS components (Figure 7C and Table S3). Moreover, the “2 ASEL” fate inducing activity of *lsy-6*, expressed from a heterologous promoter, is epistatic to the loss of COMPASS activity (*rbbp-5* mutants), corroborating the notion that COMPASS acts upstream of *lsy-6* (Figure 7D and Table S3).

Previous genetic screens have revealed that a MYST-type histone acetyltransferase (HAT) complex also shows ASE laterality defects that are indistinguishable from those of the COMPASS histone methyltransferase (HMT) complex, in that the ASEL neurons convert into ASER neurons and that *lsy-6* expression is lost in the post-mitotic ASE neurons [33]. One of the components of the MYST HAT complex was also retrieved from our RNAi screen (the PHD/Bromodomain *lin-49* locus). Given the phenotypic similarity between the MYST HAT and COMPASS HMT complex, we asked whether they genetically interact. This hypothesis was born out of biochemical studies that suggest that histone methylation is often a prerequisite for ensuing histone acetylation and transcriptional activation [34]. We tested for a genetic interaction by asking for synergies in gene function. We chose conditions in which RNAi against two different HMT components (*ash-2* and *lsy-15*) resulted in essentially no laterality defect (in a non-sensitized, rather than *nre-1; lin-15b* RNAi hypersensitive background) and tested the effect of RNAi against these genes either in a wildtype background or in a MYST HAT mutant background (the *lsy-12* MYST-like HAT gene and the *lsy-13* ING-like gene). We observed strikingly synergistic phenotypes in both *lsy-12* and *lsy-13* mutant backgrounds (Figure 7E). In contrast, *ash-2* RNAi was unable to enhance the defects of *rbbp-5* mutants (Figure 7E) and only additive or mild synergy at best was observed in *ceh-36* mutants (Table S3). All together, this data demonstrates that *rbbp-5* and the COMPASS complex acts early during embryogenesis, before the birth of the ASE neurons and in conjunction with the MYST complex to regulate the expression of the miRNA *lsy-6* and therefore ASE laterality.

## Discussion

We described here the first comprehensive, genome-wide RNAi screen that monitors a single neuron-type cell fate decision. The screen has been remarkably successful in retrieving genes known to be involved in controlling ASE neuron specification. Six genes previously known to be involved in ASE neuron specification have been found in an unbiased manner (*che-1, die-1, lsy-2, lin-49, lim-6, fozi-1*) and only two have not (*cog-1, nhr-67*: these genes also display no laterality defects with sequence-verified RNAi clones). The RNAi screen also revealed a number of genes that we would have predicted to result in ASE lineage defects, including genes involved in the segregation of early patterning cues (e.g. *par* and *mex-* genes) and involved in a re-iteratively used binary fate decision system (*lit-1* gene)[7]. Moreover, the RNAi screen assisted the cloning of two previously unknown *lsy* mutants, *lsy-15/rbbp-5* (this paper) and *lsy-22* [35] by identifying genes with laterality defects in the genetic intervals to which *lsy-15* and *lsy-22* had been mapped, facilitating the identification of the phenotype-inducing mutation within the whole-genome sequencing data.

The screen identified an as yet elusive proneural patterning gene for the ASE lineage, the bHLH gene *hlh-14*. Previous forward genetic screens for ASE differentiation mutants have failed to retrieve alleles in this gene, possibly because these screens are intrinsically biased against alleles in mutants with lethal phenotypes, illustrating a key conceptual advantage of the RNAi screening approach. Even though previous genetic screens for ASE developmental defects have successfully retrieved hypomorphic alleles of essential genes [18], in the case of *hlh-14*, alleles that separate essential functions from its proneuronal function in the ASE lineage may not exist. We have demonstrated that *hlh-14* is required for the production of neurons from several distinct lineages. Besides *lin-32/Atonal* [36], *hlh-14* is only the second bHLH gene in *C. elegans* described with such a canonical neural versus hypodermal switch function. It is possible that *hlh-14* may also regulate more general neural/non-neural decisions, such as the “non-clonal” production of neurons from the multi-potent pharyngeal lineages. Additionally, we have shown that the loss of *hlh-14* leads to neuronal sub-type differentiation defects, suggesting that, in a similar fashion to *lin-32* [37], it may act at multiple stages of neuronal differentiation.

How *hlh-14* is regulated remains to be discovered. Although early pre-patterning mechanisms that regulate proneural gene expression have been well described in both vertebrates and *Drosophila*, few direct activators of proneural genes have been identified [38], [39]. In *C. elegans*, *lin-32* is activated by Hox genes and is also repressed by *ref-1*, a downstream effector of Notch signaling [40], [41]. This suggests parallels to the Notch-mediated regulation of vertebrate proneural genes [39]. Intriguingly, an ectopic ASE lineage is generated from the ABara blastomere in *ref-1* mutants [12] and it will be interesting to determine whether this is due to a de-repression of *hlh-14*.

We observe L/R bilaterally symmetric expression of *hlh-14* in neuroblasts of symmetric origin but remarkably we also observe L/R bilaterally symmetric expression of *hlh-14* in lineages that derive from asymmetric lineage origins. This expression is initiated at an identical moment in time on both the left and right sides, despite the neuroblasts deriving from L/R asymmetric origins and is the case in both the ASEL/ASER lineages and the I1L/I1R lineages. In both cases, expression coincides with the point at which these lineages become bilaterally symmetric in that they undergo an identical set of divisions to generate the left-right homologs of bilateral pairs of neurons. Future identification of upstream regulators of *hlh-14* and other factors that are expressed during this “symmetrization phase” will begin to address how left-right bilateral symmetry is established in the nervous system. Intriguingly, we also observe left/right asymmetric expression of *hlh-14*, which is required to induce the fate of certain asymmetric neuroblasts/neurons of the C-lineage. It will be interesting to determine how this asymmetric expression is induced, how it is coordinated with bilaterally symmetric expression programs and whether, given the anterior-posterior lineage difference (Figure 6C), asymmetric expression is regulated by the binary Wnt-signaling system [7].

Given that *hlh-14*, like *lin-32*, fulfills its proneural function in several very distinct neuronal lineages [28](this paper), it is obvious that additional co-factors must exist to determine specificity of bHLH gene action. With this thought in mind, we were surprised to find essentially no other transcriptional regulator that is involved at the neuronal versus ectodermal decision step - or any later or earlier step in controlling the generation of the ASE lineage branch. This is also surprising from the perspective of the binary, Wnt-mediated a/p decision system that works broadly throughout the developing *C. elegans* embryo [7], [42], [43]. Through mutant analysis we have previously shown that this system also operates in the ASE lineage [44] and, moreover, we have also identified in this screen the *lit-1* gene, an important signal transducer of the binary system [43]. This binary specification system, whose critical output is a differential distribution of a POP-1/LEF transcription factor in dividing cells, is generally thought of as needing to pair with lineage-specific transcriptional regulators to instruct development [8], [44]. In other words, differentially distributed POP-1/LEF-1 is thought to interact with cell-intrinsic transcriptional “codes” (i.e. combinations of transcription factors) to define regulatory states that are characteristic and specific for any cell at any stage of development. Since the *pop-1* system presumably cooperates with different transcription factors at each of the a/p divisions that generate ASE, we expected to reveal a wealth of transcriptional regulators. Even if such factors had pleiotropic functions early in the embryo, our screen should have nevertheless revealed them as non-*gfp*-positive, dead embryos. Yet, with the exception of the known transcription factors (TFs) and *hlh-14*, we identified no novel TF in the set of 801 TFs screened in our RNAi library that affect ASE specification. Moreover, we noted that only about 12% of TFs in the genome-wide TF clone library resulted in the embryonic arrest phenotype that one would expect from strong lineage patterning defects. In all these arrested embryos, the ASE neurons are generated normally, with the exception of the known embryonic patterning genes *pie-1* and *skn-1*. Finally, 59 TFs generated a P0 sterile phenotype preventing the analysis of F1 phenotypes in the *nre-1 lin-15b* background. We re-screened these genes in wildtype and *rrf-3* or *eri-1; lin-15b* RNAi hypersensitive backgrounds, but uncovered no additional regulators of ASE fate (data not shown).

There are several possible and non mutually-exclusive explanations for the paucity of TF phenotypes. The simplest is a technical one, RNAi may not sufficiently knock down specific TFs. Supporting this notion, the *cog-1* homeobox gene does not display an ASE laterality defect by RNAi and was not retrieved by the screen. The other, more complex and perhaps more interesting one is well exemplified with the T box TFs *tbx-37* and *tbx-38* [13]. We have previously shown that both are required at an early point in the lineage that generates the ASEL neuron for the correct establishment of ASE asymmetry [12]. Yet both genes act redundantly; removal of either gene alone has no effect [13]. The *C. elegans* genome contains a good number of closely related, paralogous TF pairs from various families and it is conceivable that particularly during early patterning, TFs may work in redundant pairs. In addition to the *tbx-37/38* case, there are several other reported cases for such redundant TF pairs (*pes-1/fkh-2* [45], *med-1/med-2* [46]; *end-1/end-3* [47] *tbx-8/tbx-9* [48] and *ref*-family genes [41]).

The early lineage determination of ASE left-right asymmetry led us to postulate the existence of either an “asymmetry mark” that can be remembered through several rounds of division to regulate expression of the miRNA *lsy-6* expression or a cascade of TFs that leads from *tbx-37/38* to *lsy-6* [12]. Previous work from our lab has identified a MYST-type histone acetylase complex that controls ASEL/R asymmetry through regulation of the *lsy-6* miRNA [33] and in this study we have shown that the histone methyltransferase complex COMPASS also regulates ASE laterality through regulation of *lsy-6* (Figure 7). We have shown that COMPASS may be directly linked to the MYST HAT complex activity, as supported by phenotypic similarity as well as genetic interaction tests. Mechanistically, histone methylation may be a step directly upstream of and preceding histone acetylation, leading to transcriptional activation in several contexts (reviewed in [49]). In the context of ASE asymmetry, this is supported by two components of the MYST HAT complex containing a PHD domain (the LIN-49 protein and the LSY-13 protein), which is known to interact with methylated histones [34], [50]. Indeed, biochemical studies show that the PHD finger of *lsy-13* ortholog ING, recognizes the H3K4 trimethylation mark of COMPASS and serves as a link in site specific recruitment of HATs [51], [52]. Our synergistic data corroborate this idea in an *in vivo* context and demonstrate that the COMPASS complex acts before the birth of the ASE neurons, providing a potential molecular link between the early Notch signal, the expression of *tbx-37/38* and the ASEL specific expression of *lsy-6*. Together, these histone-modifying complexes may form part of a lineage-specific “chromatin mark” that acts, in cohort with specific TFs to bias *lsy-6* expression, such that it is only expressed in ASEL.

What other genes and processes has our screen revealed? Many of the genes revealed in our RNAi screen as being involved in neural development code for proteins with apparently very general cellular functions, such as general splicing factors and core components of RNA polymerases. The remarkably specific effects of some of these factors suggest that some of these proteins may not have as broad roles as previously thought or it may argue that some of the substrates (e.g. those of splicing factors) relevant to specific neuronal lineage decisions are sensitive to gene dosage (and therefore more easy to functionally impair) than other, more generally acting substrates. In addition, early *C. elegans* embryogenesis is characterized by very specific signaling events between specific individual blastomeres [53]. Several of the genes that we identified as having cell/lineage defects are likely a reflection of disrupting early blastomere divisions and positions resulting in aberrant signaling events and lineage misspecifications. For yet other genes - many of them with intriguing molecular identities - their function in determining ASE remains to be determined and will likely provide novel insights into the mechanisms of neural specification.

## Materials and Methods

### Strains and transgenes

#### Strains and transgenes used

N2 Bristol wild type [1] and CB4856 Hawaiian wild type [54]; OH2281, *rrf-3(pk1426); otIs114; him-5(e1490)*; OH7704, *eri-1(mg366); lin-15b(n744); otis114; him-5(e1490)*, and OH7082, *nre-1(hd20) lin-15b(hd126); otis114; him-5(e1490)* [20]. VC1137, *hda-1(ok1595) V/nT1[qIs51]* (IV;V); VC954, *rnf-113(ok1401) III/hT2[bli-4(e937) let-?(q782) qIs48]*(I;III); JK907, *mog-4(q233)/mnC1 dpy-10(e128) unc-52(e444)*II; VC2127, *mog-4(ok2708)/mT1* II; *+/mT1[dpy-10(e128)]* III; JK2321, *mog-5(q449) unc-4(e120)/mIn1[dpy-10(e128)]* II; VC724, *mog-5(ok1101)/mIn1[mIs14 dpy-10(e128)]* II; RB1593, *klp-15(ok1505)* I; RW3199, *lev-11(x12) let-49(st44)/lev-11(x12) unc054(e1152)* I; VC186 *smo-1(ok359) I/szT1 [lon-2(e678)* (I;X); NG2295, *hlh-14(gm34)/mnC1 dpy-10(e128) unc-52(e444)* II [28]; NG4280, *hlh-14(tm295)/mIn1 [mIs14 dpy-10(e128)]* II [28]; RB979, *F28B3.1(ok885)* I; SM1017, *tlf-1(ok389)/unc-55(e402) dpy-24(s71)* I; KR499, *let-526(h185) dpy-5(e61) unc-13(e450)* I; *sDp2(I;f); VC1395, pqn-38(ok1791) III/hT2[bli-4(e937) let-?(q782) qIs48]* (I;III); RB1025, *set-2(ok952)* III; *MT14851, set-2(n4589)* III; RB1304 *swd-3.1(ok1417). dpy-30(y228) and dpy-30(y130)* III courtesy of B.J. Meyer. *F21H12.1(tm3463)* and *F21H12.1(tm3530)* II, *grp-1(tm1956)* III, *ash-2(tm1905)* II, *mag-1(tm645)* I: all obtained from Mitani Laboratory, NBP, Japan. *lsy-15(ot86)* was isolated as previously described [18].

#### Transgenes to label ASEL and ASER fates

*otIs3 V, Is[gcy-7^prom^::gfp; lin-15 (+)]; otIs114* I, Is*[lim-6^prom^::gfp; rol-6(d)]; otIs220* IV, Is*[gcy-5^prom^::mCherry; rol-6(d)]; otIs186* Is*[gcy-5^prom^::gfp]; ntIs1* V, Is*[gcy-5^prom^::gfp; lin-15 (+)]*; and the ASEL/ASER markers otIs151 V, Is*[ceh-36^prom^::DsRed2; rol-6(d)]* and otIs232 V, Is*[che-1^prom^::mCherry; rol-6(d)]*, otIs217 Is*[che-1^prom^::HIS-3::mCherry; rol-6(d)]* and otIs299 Is*[8xASE::gfp; elt-2::dsRed]*.

#### Reporters for other cell types

OLL *otIs138* Is[*ser-2^prom3^::gfp; lin-15(+)*] [55], AFD *oyIs17* Is[*gcy-8^prom^::gfp; lin-15(+)*] [56]; muscle *ccIs4251*, Is[*myo-3^prom^::DFP-LacZ(NLS); myo-3^prom^::mitochondrial GFP; dpy-20(+)*] [4]; gut *(elt-2::gfp/LacZ)* (personal communication A. Grishok); dopaminergic and cholinergic neurons *otIs181*, Is[*dat-1^prom^::mCherry; ttx-3^prom^::mCherry*] [57]; hypodermis *arIs99*, Is[*dpy-7^prom^::2Xnls::yfp; ceh-22::gfp*] [58]; pan-neuronal *otIs287*, *Is[rab-3^prom^::nls::yfp]; gmIs20* Is[*hlh-14^prom^::gfp*] [28].

#### Fosmid recombineering and transgenic arrays

Fosmids were tagged as described [29] to generate OH10173-OH10176 *otEx4507-otEx45010*, Ex[*hlh-14^FOS^(WRM0627dH07)::yfp; rol-6(d)*]; *hlh-14(tm295)*; *ntIs1* (4 independent lines); OH10171 *otEx4506*, Ex[*rbbp-5^FOS^(WRM064bD12)::gfp*; *ttx-3^prom^::mCherry*]; *otIs232*.

Rescue of *lsy-15(ot86)*: In addition to the tagged fosmid line (above) the following untagged fosmid arrays were generated: OH9335-OH9336, *otEx4135-otEx4136 Ex[F21H12.1^FOS^ (WRM061bG10);elt-2::gfp]* line 1 (2 independent lines).

Rescue of *lsy-15(tm3463)*: Heterologous promoter constructs were made by PCR fusion ([59]; primers available upon request) to generate OH10177-OH10179 *otEx4511-otEx4513*, Ex[*che-1^prom^::rbbp-5::gfp; elt-2^prom^::gfp*] (3 independent lines); OH10180-OH10182 *otEx4514-otEx4516*, Ex[*hsp-16^prom^::rbbp-5::gfp; elt-2^prom^::gfp*] (3 independent lines; 50 ng/ul); OH10183-OH10185 *otEx4517-otEx4519*, Ex[*hsp-16^prom^::rbbp-5::gfp; elt-2^prom^::gfp*] (3 independent lines; 20 ng/ul); OH10186-OH10188 *otEx4520-otEx4522*, Ex[*hsp-16^prom^::rbbp-5::gfp; elt-2^prom^::gfp*] (3 independent lines; 2 ng/ul).

Overexpression: OH7805 *otIs204* Is*[ceh-36^prom2^::lsy-6, elt-2^prom^::gfp]*.

### RNA interference

RNAi was performed using a bacterial feeding protocol [23] with the following modifications. NGM agar plates containing 6 mM IPTG and 100 µg/ml ampicillin were seeded with bacteria expressing dsRNA. L4 staged hermaphrodites were placed onto these plates and grown at 22°C. After five days, the F1 progeny of these worms were scored for neuronal and pleiotropic defects. All inserts of clones for the known ASE factors were sequenced to confirm identity of the clone. The visible phenotypes screened for chromosome I: Emb (embryonic lethal), P0 Ste (P0 sterile), Stp (sterile progeny), Brd (low brood size), Gro (slow postembryonic growth), Lva (larval arrest), Let (larval lethal), Adl (adult lethal), Bli (blistering of cuticle), Bmd (body morphological defects), Clr (clear), Dpy (dumpy), Egl (egg- laying defective), Lon (long), Mlt (molt defects), Muv (multivulva), Prz (paralyzed), Pvl (protruding vulva), Rol (roller), Rup (ruptured), Sck (sick) and Unc (uncoordinated). Emb was defined as greater than 30% dead embryos. Each phenotype was required to be present among at least 10% of the analyzed worms for the visible pleiotropies and neuronal defects. Non-viable (nonV) category encompasses P0 Ste, Emb, Stp and Let phenotypes. The JA library of RNAi clones was purchased from Geneservice (http://www.lifesciences.sourcebioscience.com/) and was supplemented with the ORFeome based RNAi clones [60] courtesy of John Kim [61]. Based on sequencing of several randomly picked clones from the library we estimate the faithfulness of the library to be 80–90% (data not shown).

### Phenotypic scoring criteria

#### Primary screen

Primary screen was conducted by screening two wells for the phenotypic categories “loss of ASEL” and “ectopic ASEL”. A positive clone was defined as having a phenotype in at least 3/8 wells scored (or > = 0.4). If clone had a phenotype (at least one well), it was then re-screened an additional 6 times (see below). An ASEL loss score was generated by dividing the number of wells showing “loss of ASEL” over the total number of wells scored. An ASEL ectopic score was generated by dividing the number of wells showing “ectopic ASEL” over total number of wells scored. Thus, in Tables 1, 2, 3, 4 and Tables S1 and S2 (see Results):

ASEL off (-)  =  ASEL off score >0, ASEL ectopic score 0;

ASEL ectopic (+)  =  ASEL ectopic score >0, ASEL off score 0;

ASEL mixed (+/−)  =  score in both ASEL off and ASEL ectopic >0. For ASEL mixed category the following adjustment was made: based on the ratio of (ASEL ectopic/ASEL off score) if score < = 0.25 clones were moved to ASEL off. Based on ratio (ASEL off/ASEL ectopic score) if score < = 0.25 clones were moved to ASEL ectopic.

#### The ASE screen

The ASE screen was conducted by screening 6 wells with an ASER specific reporter and bilateral ASE reporter. Phenotypic scoring for ASER reporter consisted of “loss of ASER” and “ectopic ASER” while for bilateral ASE reporter was “off”, “1-on”, “2-on”, “ectopic ASEs” and “mixed” where “mixed” category was any combination of the above phenotypes. The ASER phenotypic categorization was generated in the same way as the ASEL above. Thus, in Tables 1, 2, 3, 4 and Tables S1 and S2 (see Results):

ASER off (-)  =  ASER off score >0, ASER on score 0, > = 1 wells screened;

ASEL ectopic (+)  =  ASER on score >0, ASER off score 0, > = 1 wells screened;

ASER mixed (+/−)  =  ASER off score >0, ASER ectopic score >0, > = 1 wells screened;

ASER wt  =  ASER score  =  0, > = 1 wells screened.

Blank means not done.

The ASE bilateral score was generated as follows:

ASE wt  =  ASE score  =  0, > = 1 wells screened;

ASE 2-off (-)  =  ASE 2-off score >0, all other scores 0, > = 1 wells screened;

ASE 1-off (-)  =  ASE 1-off score >0, all other scores 0, > = 1 wells screened;

ASE ectopic (+)  =  ASE ectopic score >0, all other scores 0, > = 1 wells screened;

ASE mixed (+/−)  =  ASE off scores (either of two) >0, ASE ectopic score >0; or if scored >0 in the ASE “mixed”, > = 1 wells screened.

#### The tissue marker screen

The tissue marker screen was conducted by screening two wells, for phenotypic categories “tissue OFF” “tissue Mixed” “tissue ON”, which were assigned an arbitrary score of 0, 0.25 and 0.5 respectively. The final tissue score of the clone was calculated by adding the individual scores of the two wells, resulting in a range from minimum 0 (0+0) to maximum 1 (0.5+0.5). If the score >0.5, the clone is “ON” for that tissue; if the score is < = 0.5, the clone is designated “OFF”. In the Tables 1, 2, 3, 4 and Tables S1 and S2 (see Results Section) “ON” denoted “+”, OFF denoted “−”.

“ne” means clone not embryonic lethal. Clones that were “ne” in 3 or more strains (out of 6 strains screened) were taken off the list under the premise of non-repeatable embryonic phenotype. Categorization in groups is described in Results.

### Lineage and 3D cell position analysis of *hlh-14* expression and *hlh-14* mutants

Cells were identified using 4D microscopy and SIMI BioCell software as described in [10]. Briefly, wild-type (containing the *ntIs1* transgene), *hlh-14:gfp (gmIs20)*, *hlh-14(gm34); otIs114*, *hlh-14(tm295); ntIs1; otEx4507 Ex[hlh-14^FOS^::YFP]* gravid adults were dissected and single two-cell embryos were mounted and visualized on a Zeiss Axioplan 2 compound microscope. To lineage *hlh-14(tm295)* mutants, non-array carrying embryos from the *hlh-14(tm295); ntIs1; otEx4507 Ex[hlh-14^FOS^::YFP]* strain were examined. Nomarski stacks were taken every 35 seconds and images within a stack at ∼1 µm apart. Fluorescent images were acquired at pre-determined time points during the course of embryonic development. Movies were lineaged using the SIMI BioCell program. The positions of cells at different time-points were monitored using the 3D-feature of SIMI BioCell. All additional DIC and fluorescent images were acquired using the open source Micro-manager software [62].

### Heat-shock *rbbp-5* rescue experiments

Embryos were dissected from array carrying mothers, staged and then subjected to a 15 minute heat-shock at 37°C after which the embryos were placed at 15°C and allowed to develop to the L1 larval stage before scoring. Embryos were divided into two categories for heat-shocks: ‘early’  =  younger than AB^256^-cell stage; ‘late’  =  older than AB^256^-cell stage.

### Mapping and whole-genome sequencing

The *lsy-15(ot86)* allele was mapped to a region of approximately 15 MB (Figure 6) through classic three-factor and single-nucleotide polymorphism (SNP) mapping. Then, genomic DNA was prepared from a population of *lsy-15(ot86); otIs114* animals using the Gentra GenePure DNA extraction kit. This genomic DNA (5 µg) was genome sequenced as previously described [63] using an Illumina Genome Analyzer II according to the manufacturers specification. The resultant sequence data was analyzed with MAQGene [64]. The following stringency criteria were used to filter variants: quality score > = 3, loci multiplicity < = 1, sequencing depth > = 3. All detected sequence variants were analyzed for whether they were present in any one of several additional genomes of different genotypes that we sequenced in the laboratory; if so, they were considered background and eliminated from further analysis.

## Supporting Information

Figure S1 Phenotypic classification in our screen for clones previously reported to generate a Gro phenotype. Pie-chart that illustrates the phenotype we observed in our screen for the 303 RNAi clones previously reported to generate a slow growing (Gro) phenotype [22], [23].
(EPS)

Figure S2 Only *mex-3* and *mex-5* generate pan-neuronal differentiation defects. RNAi of those genes we identified in the tissue screen as generating broad neuronal differentiation defects in an non-sensitized strain carrying a pan-neuronal reporter (*rab-3::yfp*). Indicated in each panel is the number of embryos that showed a reduced number of *rab-3* positive cells. In all cases only those embryos that were lethal were examined, but we note that in all cases the embryonic lethality was >50% and in most cases close to 100%. No genes, other than *mex-3* and *mex-5* lead to a near complete loss of all neurons. The paralogous G-protein regulators *gpr-1* and *gpr-2* led to a mild but identifiable loss of many neurons suggesting very broad defects, although the arrest stage of embryos was somewhat variable. The phosphatase *T09A5.9* led to a low penetrant but identifiable loss of *rab-3::yfp* expression suggesting it is the next broadest.
(EPS)

Figure S3 Ectopic expression of the *dpy-7* hypodermal marker in *hlh-14(tm295)* mutants. Expression of *dpy-7::2Xnls::yfp* can be easily observed in ectopic hyp7 cells in the posterior of the embryo and in an ectopic hyp11 cell (out of focus in the images). In the anterior of the embryo ectopic or mispositioned hyp cells can be observed (asterisks). Several embryos from an *hlh-14(tm295)/mIn1* mother were scored, defects were observed in approximately 25% of these embryos as expected. The total number of embryos displaying the defects is indicated beneath the figure.
(EPS)

Figure S4 Bilaterally symmetric expression of *hlh-14* in other neuronal lineages. A: Ventral view of the 3D-poitions of additional bilaterally symmetric cells that express *hlh-14* at the AB^256^-cell stage. In the posterior of the embryo neuroblasts from the predominantly hypodermal ABplap/prap (green) and ABarpp (pink) lineages express *hlh-14*. Cell coloring after [10]. B: Panel shows the expression (purple lines) of *hlh-14* and in certain cases the division pattern as determined by 4D-lineage analysis of the ABplap/prap lineages of wild-type embryos (containing the *ntIs1* transgene). *hlh-14* expression was assessed in both *gmIs20* and *hlh-14(tm295); Ex[hlh-14^FOS^::yfp]* animals. In the case of the PLM/ALN lineage, expression was only observed in *gmIs20*. Red cell names indicate neurons/glia and yellow cell names indicate hypodermis. C: Panel shows the expression (purple lines) of *hlh-14* in the ABarpp lineage in wild-type embryos, as determined by 4D-lineage analysis. Red cell names indicate neurons/glia and yellow cell names indicate hypodermis.
(EPS)

Figure S5 Bilaterally symmetric expression and function of *hlh-14* in the I1L/I1R lineage. A: Ventral view of the 3D-poitions of the bilaterally symmetric I1L/I1R neuroblasts (light blue) at the AB^256^-cell stage. The gut lineage is shown for positional information. Cell coloring after [10]. B: Lineage and *hlh-14* expression analysis of the I1L/I1R lineage in wildtype (containing the *ntIs1* transgene), *hlh-14(tm295); ntIs1* and *hlh-14(tm295); otEx4507,* Ex*[hlh-14^FOS^::yfp]; ntIs* embryos. Purple lines indicate the expression of *hlh-14* (assessed in *hlh-14(tm295); Ex[hlh-14^FOS^::yfp* embryos) and the numbers below indicate the number of lineages that divided as indicated and the total number of left or right lineages followed to terminal division are shown at the bottom of each lineage diagram indicate the total number of lineages followed. The mother of the I1L/I1R blastomeres divides early in *hlh-14* mutants, at a time when sister lineage divides to produce one pharyngeal marginal cell (mc1) and one pharyngeal muscle cell (m3V). This defect is rescued with the *hlh-14^FOS^::yfp* transgene. Red cell names indicate neurons/glia and green cell names indicate mesoderm.
(EPS)

Figure S6 Expression of *che-1^prom^::rbbp-5::gfp* and *hsp-16^prom^::rbbp-5::gfp* transgenes. The *che-1* promoter driven *rbbp-5::gfp* translational fusion is brightly expressed in ASE but does not rescue the *rbbp-5(tm3463)* 2-ASER phenotype (see Figure 6). At room temperature leaky expression of the heat-shock promoter driven *rbbp-5(tm3463)::gfp* transgene can be observed in many cells, but rarely in the ASE neurons, yet this rescues the *rbbp-5(tm3463)* 2-ASER phenotype (see Figure 6).
(EPS)

Table S1 Genes that elicit a mixed ASE fate when knocked-down by RNAi. See Materials and Methods for a detailed description of scoring criteria. Abbreviations: ne, not embryonic lethal; wt, wildtype.
(XLSX)

Table S2 Genes that elicit an early/cell lethal mixed phenotype when knocked-down by RNAi. See Materials and Methods for a detailed description of scoring criteria. Abbreviations: ne, not embryonic lethal; wt, wildtype.
(XLSX)

Table S3 COMPASS complex genes regulate ASE asymmetry upstream of the microRNA *lsy-6.* Detailed scoring of COMPASS complex component RNAi or mutant animals. ^1^ASEL fate scored with *lim-6::gfp(otIs114).* ^2^ASER fate scored with *gcy-5^prom^::gfp(ntIs1), **gcy-5^prom^::rfp (otIs220) or ***gcy-5^prom^::gfp(otIs186). ^3^Wildtype phenotype for all three ASER reporter strains is 100% intact. ^4^Scored as young larvae. ^5^ *ASE::lsy-6* is *ceh-36^prom^::lsy-6 (otIs204)*. ^6^Ex[*lsy-6::yfp*] is an extrachromosomal array, *otEx4410* Ex[*lsy-6::yfp; ttx-3::mcherry*]. Only array carrying worms were scored. ^7^For the ASEL scoring of *ash-2* and *rbbp-5* RNAi in a wildtype backgroud, RNAi was performed in both a strain containing the *lim-6::gfp(otIs114)* reporter to match the reporter present in *lsy-12(ot154)* strain and in a strain containing both the *lim-6::gfp(otIs114)* and *gcy-5::rfp(otIs220)* reporter to match the *lsy-13(ok1417)* strain. No difference was observed and the numbers were pooled.
(XLSX)

## References

[1] Brenner S (1974). The genetics of Caenorhabditis elegans.. Genetics.

[2] Wood WB (1988). The nematode Caenorhabditis elegans: Cold Spring Harbor Laboratory Press.

[3] Hobert O (2010). Neurogenesis in the nematode Caenorhabditis elegans.. WormBook.

[4] Fire A, Xu S, Montgomery MK, Kostas SA, Driver SE (1998). Potent and specific genetic interference by double-stranded RNA in Caenorhabditis elegans [see comments].. Nature.

[5] Kamath RS, Ahringer J (2003). Genome-wide RNAi screening in Caenorhabditis elegans.. Methods.

[6] Sulston JE, Schierenberg E, White JG, Thomson JN (1983). The embryonic cell lineage of the nematode Caenorhabditis elegans.. Dev Biol.

[7] Bertrand V, Hobert O (2010). Lineage programming: navigating through transient regulatory states via binary decisions.. Curr Opin Genet Dev.

[8] Lin R, Hill RJ, Priess JR (1998). POP-1 and anterior-posterior fate decisions in C. elegans embryos.. Cell.

[9] Sulston JE (1983). Neuronal cell lineages in the nematode Caenorhabditis elegans.. Cold Spring Harb Symp Quant Biol.

[10] Schnabel R, Hutter H, Moerman D, Schnabel H (1997). Assessing normal embryogenesis in Caenorhabditis elegans using a 4D microscope: variability of development and regional specification.. Dev Biol.

[11] Ortiz CO, Faumont S, Takayama J, Ahmed HK, Goldsmith AD (2009). Lateralized gustatory behavior of C. elegans is controlled by specific receptor-type guanylyl cyclases.. Curr Biol.

[12] Poole RJ, Hobert O (2006). Early embryonic programming of neuronal left/right asymmetry in C. elegans.. Curr Biol.

[13] Good K, Ciosk R, Nance J, Neves A, Hill RJ (2004). The T-box transcription factors TBX-37 and TBX-38 link GLP-1/Notch signaling to mesoderm induction in C. elegans embryos.. Development.

[14] Johnston RJ, Hobert O (2003). A microRNA controlling left/right neuronal asymmetry in Caenorhabditis elegans.. Nature.

[15] Chang S, Johnston RJ, Hobert O (2003). A transcriptional regulatory cascade that controls left/right asymmetry in chemosensory neurons of C. elegans.. Genes Dev.

[16] Johnston RJ, Chang S, Etchberger JF, Ortiz CO, Hobert O (2005). MicroRNAs acting in a double-negative feedback loop to control a neuronal cell fate decision.. Proc Natl Acad Sci U S A.

[17] Didiano D, Cochella L, Tursun B, Hobert O (2010). Neuron-type specific regulation of a 3′UTR through redundant and combinatorially acting cis-regulatory elements.. RNA.

[18] Sarin S, O'Meara M M, Flowers EB, Antonio C, Poole RJ (2007). Genetic Screens for Caenorhabditis elegans Mutants Defective in Left/Right Asymmetric Neuronal Fate Specification.. Genetics.

[19] Fire A (1999). RNA-triggered gene silencing.. Trends Genet.

[20] Schmitz C, Kinge P, Hutter H (2007). Axon guidance genes identified in a large-scale RNAi screen using the RNAi-hypersensitive Caenorhabditis elegans strain nre-1(hd20) lin-15b(hd126).. Proc Natl Acad Sci U S A.

[21] Kennedy S, Wang D, Ruvkun G (2004). A conserved siRNA-degrading RNase negatively regulates RNA interference in C. elegans.. Nature.

[22] Simmer F, Moorman C, Van Der Linden AM, Kuijk E, Van Den Berghe PV (2003). Genome-Wide RNAi of C. elegans Using the Hypersensitive rrf-3 Strain Reveals Novel Gene Functions.. PLoS Biol.

[23] Kamath RS, Fraser AG, Dong Y, Poulin G, Durbin R (2003). Systematic functional analysis of the Caenorhabditis elegans genome using RNAi.. Nature.

[24] Zipperlen P, Fraser AG, Kamath RS, Martinez-Campos M, Ahringer J (2001). Roles for 147 embryonic lethal genes on C.elegans chromosome I identified by RNA interference and video microscopy.. Embo J.

[25] Ciosk R, DePalma M, Priess JR (2006). Translational regulators maintain totipotency in the Caenorhabditis elegans germline.. Science.

[26] Watts JL, Etemad-Moghadam B, Guo S, Boyd L, Draper BW (1996). par-6, a gene involved in the establishment of asymmetry in early C. elegans embryos, mediates the asymmetric localization of PAR-3.. Development.

[27] Uchida O, Nakano H, Koga M, Ohshima Y (2003). The C. elegans che-1 gene encodes a zinc finger transcription factor required for specification of the ASE chemosensory neurons.. Development.

[28] Frank CA, Baum PD, Garriga G (2003). HLH-14 is a C. elegans achaete-scute protein that promotes neurogenesis through asymmetric cell division.. Development.

[29] Tursun B, Cochella L, Carrera I, Hobert O (2009). A toolkit and robust pipeline for the generation of fosmid-based reporter genes in C. elegans.. PLoS ONE.

[30] Tenney K, Shilatifard A (2005). A COMPASS in the voyage of defining the role of trithorax/MLL-containing complexes: linking leukemogensis to covalent modifications of chromatin.. J Cell Biochem.

[31] Simonet T, Dulermo R, Schott S, Palladino F (2007). Antagonistic functions of SET-2/SET1 and HPL/HP1 proteins in C. elegans development.. Dev Biol.

[32] Xu L, Strome S (2001). Depletion of a novel SET-domain protein enhances the sterility of mes-3 and mes-4 mutants of Caenorhabditis elegans.. Genetics.

[33] O'Meara MM, Zhang F, Hobert O (2010). Maintenance of Neuronal Laterality in Caenorhabditis elegans Through MYST Histone Acetyltransferase Complex Components LSY-12, LSY-13 and LIN-49.. Genetics.

[34] Taverna SD, Ilin S, Rogers RS, Tanny JC, Lavender H (2006). Yng1 PHD finger binding to H3 trimethylated at K4 promotes NuA3 HAT activity at K14 of H3 and transcription at a subset of targeted ORFs.. Mol Cell.

[35] Flowers EB, Poole RJ, Tursun B, Bashllari E, Pe'er I (2010). The Groucho ortholog UNC-37interacts with the short Groucho-like protein LSY-22 to control developmental decisions in C. elegans.. Development.

[36] Zhao C, Emmons SW (1995). A transcription factor controlling development of peripheral sense organs in C. elegans.. Nature.

[37] Portman DS, Emmons SW (2000). The basic helix-loop-helix transcription factors LIN-32 and HLH-2 function together in multiple steps of a C. elegans neuronal sublineage.. Development.

[38] Skeath JB, Carroll SB (1994). The achaete-scute complex: generation of cellular pattern and fate within the Drosophila nervous system.. The FASEB journal: official publication of the Federation of American Societies for Experimental Biology.

[39] Bertrand N, Castro DS, Guillemot F (2002). Proneural genes and the specification of neural cell types.. Nat Rev Neurosci.

[40] Ross JM, Kalis AK, Murphy MW, Zarkower D (2005). The DM domain protein MAB-3 promotes sex-specific neurogenesis in C. elegans by regulating bHLH proteins.. Developmental cell.

[41] Neves A, Priess JR (2005). The REF-1 family of bHLH transcription factors pattern C. elegans embryos through Notch-dependent and Notch-independent pathways.. Dev Cell.

[42] Sawa H (2010). Specification of neurons through asymmetric cell divisions.. Curr Opin Neurobiol.

[43] Kaletta T, Schnabel H, Schnabel R (1997). Binary specification of the embryonic lineage in Caenorhabditis elegans.. Nature.

[44] Bertrand V, Hobert O (2009). Linking asymmetric cell division to the terminal differentiation program of postmitotic neurons in C. elegans.. Dev Cell.

[45] Molin L, Mounsey A, Aslam S, Bauer P, Young J (2000). Evolutionary conservation of redundancy between a diverged pair of forkhead transcription factor homologues.. Development.

[46] Maduro MF, Meneghini MD, Bowerman B, Broitman-Maduro G, Rothman JH (2001). Restriction of mesendoderm to a single blastomere by the combined action of SKN-1 and a GSK-3beta homolog is mediated by MED-1 and -2 in C. elegans.. Molecular cell.

[47] Maduro MF, Rothman JH (2002). Making worm guts: the gene regulatory network of the Caenorhabditis elegans endoderm.. Dev Biol.

[48] Pocock R, Ahringer J, Mitsch M, Maxwell S, Woollard A (2004). A regulatory network of T-box genes and the even-skipped homologue vab-7 controls patterning and morphogenesis in C. elegans.. Development.

[49] Latham JA, Dent SY (2007). Cross-regulation of histone modifications.. Nat Struct Mol Biol.

[50] Vezzoli A, Bonadies N, Allen MD, Freund SM, Santiveri CM (2010). Molecular basis of histone H3K36me3 recognition by the PWWP domain of Brpf1.. Nat Struct Mol Biol.

[51] Martin DG, Baetz K, Shi X, Walter KL, MacDonald VE (2006). The Yng1p plant homeodomain finger is a methyl-histone binding module that recognizes lysine 4-methylated histone H3.. Mol Cell Biol.

[52] Pena PV, Davrazou F, Shi X, Walter KL, Verkhusha VV (2006). Molecular mechanism of histone H3K4me3 recognition by plant homeodomain of ING2.. Nature.

[53] Schnabel R, Priess JR, Riddle DL, Blumenthal T, Meyer BJ, Priess JR (1997). Specification of Cell Fates in the Early Embryo.. Celegans II.

[54] Hodgkin J, Doniach T (1997). Natural variation and copulatory plug formation in Caenorhabditis elegans.. Genetics.

[55] Tsalik EL, Niacaris T, Wenick AS, Pau K, Avery L (2003). LIM homeobox gene-dependent expression of biogenic amine receptors in restricted regions of the C. elegans nervous system.. Dev Biol.

[56] Lanjuin A, VanHoven MK, Bargmann CI, Thompson JK, Sengupta P (2003). Otx/otd Homeobox Genes Specify Distinct Sensory Neuron Identities in C. elegans.. Dev Cell.

[57] Flames N, Hobert O (2009). Gene regulatory logic of dopamine neuron differentiation.. Nature.

[58] Myers TR, Greenwald I (2005). lin-35 Rb acts in the major hypodermis to oppose ras-mediated vulval induction in C. elegans.. Developmental cell.

[59] Hobert O (2002). PCR fusion-based approach to create reporter gene constructs for expression analysis in transgenic C. elegans.. Biotechniques.

[60] Rual JF, Ceron J, Koreth J, Hao T, Nicot AS (2004). Toward improving Caenorhabditis elegans phenome mapping with an ORFeome-based RNAi library.. Genome Res.

[61] Kim JK, Gabel HW, Kamath RS, Tewari M, Pasquinelli A (2005). Functional genomic analysis of RNA interference in C. elegans.. Science.

[62] Edelstein A, Amodaj N, Hoover K, Vale R, Stuurman N (2010).

[63] Sarin S, Prabhu S, O'Meara MM, Pe'er I, Hobert O (2008). Caenorhabditis elegans mutant allele identification by whole-genome sequencing.. Nat Methods.

[64] Bigelow H, Doitsidou M, Sarin S, Hobert O (2009). MAQGene: software to facilitate C. elegans mutant genome sequence analysis.. Nat Methods.

